# Modeling Geneva charitable deductions: regular giving and future trends

**DOI:** 10.1007/s10260-025-00828-7

**Published:** 2026-01-19

**Authors:** Marta Pittavino, Giedre Lideikyte-Huber

**Affiliations:** 1 Venice School of Management (VSM), Ca’ Foscari University of Venice (UNIVE), Venice, Italy; 2 Academic Fellow, Geneva Centre for Philanthropy (GCP), University of Geneva (UNIGE), Geneva, Switzerland; 3 Institute for Law and Economics, University of St. Gallen, St. Gallen, Switzerland

**Keywords:** Wealth tax incentives, Regularity, Regression methods, Time-series forecasts, ETS and ARIMA models

## Abstract

This study conducts the first large-scale analysis of tax incentives for charitable giving in Switzerland, focusing on regular donors and using innovative statistical forecasts. For the first time, these estimates are validated with cumulative data from Geneva authorities. Using unique panel data from Geneva taxpayers (2001–2011), this study examines income tax deductions, donation frequency, and donor characteristics, alongside forecasts for future charitable deductions and donor trends. This period also included a legal reform that raised the deduction ceilings. Findings reveal that 29% of donors deducted only once, while 71% deducted multiple times. Regular donors are not significantly influenced by tax incentive ceilings, but their donations increase over time. Wealth plays a crucial role in donation frequency, as shown by linear regression estimates. Time-series forecasting models predict total deductions steadily reaching CHF135 million by 2022, with results validated against actual data. The donor population was also forecasted to grow, reaching 72,349 by 2022, four times the 2001 figure. Based on these empirical findings, the study suggests policy measures to enhance charitable giving, aligning with legislative tax reforms. These projections offer valuable insights into optimizing tax incentives.

**Supplementary Information:**

The online version contains supplementary material available at 10.1007/s10260-025-00828-7.

## Introduction

Tax incentives for charitable giving are a common feature of tax systems worldwide (*OECD, *2020). Their stated economic aim is to encourage donations, though other objectives—such as promoting transparency in the philanthropic sector—are also often cited (Reiser and Dean 2023). Yet legislative reforms in this area usually remain vague: while they stress the goal of boosting charitable giving, they rarely specify how taxpayers are expected to respond, which types of donors are targeted, or how effectiveness will be assessed (Lideikyte-Huber, Pittavino, and Peter 2021).

This lack of precision is striking in light of broader debates on wealth inequality and redistribution. Rising disparities in income and wealth have revived interest in how private fortunes might be mobilized for public purposes (Piketty and Saez 2003; Piketty and Zucman 2014; Gravelle 2020). At the same time, some governments have been retreating from direct support for nonprofit sectors, shifting greater responsibility to private philanthropy. In the United States, for example, the Trump administration drastically reduced USAID funding, scaling back the government’s role in development and humanitarian assistance (Reuters 2025a, b). Such developments make it increasingly urgent to understand what motivates individuals to give and how tax incentives can shape their behavior.

Despite this urgency, empirical research on donor behavior—especially among higher-income and wealthier taxpayers—remains limited, particularly in Europe. U.S. studies show a tendency toward increasing concentration of philanthropy among high-wealth households (Duquette 2018; Duquette and Mayo 2021), but comparable European evidence is scarce. Moreover, relatively few studies examine the regularity of donors who respond to tax incentives, and even fewer investigate sustained changes in behavior following tax law reforms. One of the most significant recent contributions, by Ring and Thoreson (2021, 2022, 2025), analyzes the effect of wealth taxation on charitable giving. However, their work does not address donation regularity or the interaction of wealth and income in shaping donor behavior.

Part of the challenge lies in the data. Reliable information on private wealth is seldom available, in part because wealth taxation itself has become increasingly rare, now applied in only four OECD countries (OECD 2018). Switzerland is a rare exception. Its cantons levy a wealth tax and collect detailed administrative records. Yet even here, authorities rarely grant researchers access to such information.

The panel dataset we use is indeed rare in several respects. First, it provides access to both income and wealth data at the individual taxpayer level, which is seldom available due to strict data protection and confidentiality rules. Second, the dataset covers an unusually long period (2001–2011), enabling a robust longitudinal analysis of donor behavior over time. Third, it includes administrative data for the entire population of taxpayers in the Canton of Geneva, not just a sample, which enhances the representativeness and accuracy of the findings. These features combined allow us to analyze donation behavior with a level of detail and precision that is rarely achievable in studies of charitable giving.

The objective of this study is threefold. First, we examine the behavior of regular donors in Geneva, focusing on the frequency and continuity of their deductions across an extended period. Second, we analyze the relative importance of income and wealth in explaining donor regularity, distinguishing between those who consistently donate and those who seek to maximize deduction ceilings. Third, we apply time-series forecasting techniques to predict future trends in both deduction amounts and donor numbers, validating these forecasts against actual cumulative tax data.

By combining legal context, empirical evidence, and statistical modeling, our study contributes to a more precise understanding of how tax incentives interact with wealth and donor behavior. The findings also carry direct relevance for policymakers seeking to design effective tax measures that sustain and expand charitable giving.

The remainder of this paper is structured as follows: Sect. 2 outlines the Swiss tax law framework, Sect. 3 describes the data and methods, Sect. 4 presents the empirical results, and Sect. 5 concludes.

## Swiss tax law framework

Current Swiss law allows taxpayers to deduct, up to a certain threshold, charitable contributions from their taxable income for individuals and corporations. This deduction is subject to a threshold which is 20% of the net taxable income or profits for federal income tax purposes, with a minimum donation requirement of CHF 100 (*Federal Act of 14 December 1990 on Direct Federal Taxation (DFTA), AS 1991 1184, Art. 33a DFTA*). According to the Federal Constitution, cantons can set their own income tax rates, but most of the cantonal thresholds are also set at 20% (*Art. 129 of the Swiss Federal Constitution*). It is not possible to deduct donations to political parties under this norm; membership contributions and other payments of up to CHF 10,300 to a political party are deductible as general deductions under another legal norm (*Art. 33 para. 1(i) DFTA*).

The deductible donation must be paid to a legal entity that receives a tax exemption for pursuing public interest or service aims (*Arts 33a and 56 let. g DFTA*). The conditions for this type of entity's tax exemption are laid out in legislation and in case law. One of the primary requirements is that the entity cannot pursue economic aims since they cannot be regarded as being in the interest of the general public (*Art. 56 let. g DFTA*). For instance, maintaining sizable shareholdings in business firms is, in principle, considered an economic goal; it can only be accepted if it is subordinate to the entity's pursuit of public interest goals, and if it is necessary to achieve them.

Tax laws changed the ceiling and conditions for charitable deductions twice during the study period: at the cantonal level in 2001 and 2009 respectively. In our study, we sought to understand whether these changes had an impact on deduction behavior.

The first reform came into force at the cantonal level in 2001, at the very beginning of our study period. It introduced the possibility of deducting donations made not only to charitable organizations established in the canton of Geneva but also to those established elsewhere in Switzerland (Lideikyte-Huber and Peter 2022). In addition, the rule on deductions has been simplified, and the circle of eligible charities has been widened: before 2001, only religious, social, humanitarian, cultural or nature conservation objectives were considered eligible (Lideikyte-Huber and Peter 2022).

The second cantonal reform followed the changes that had been carried out at the federal level. On January 1, 2006, the federal legislator introduced the 20% deduction threshold on taxable income as part of a larger reform of the Swiss federal law (*Swiss civil code—modifications, Code civil suisse* 2004)), replacing the previous threshold of 10%. This reform also carried out other major modifications of federal tax law norms related to charitable giving, introducing a deduction for charitable non-cash donations and allowing the deduction of donations to the Swiss Confederation, the cantons, the communes, and their institutions (*Swiss civil code—modifications, The Federal Constitution of the Swiss Confederation* 1999). The general aim of this reform was to encourage donors “to give up part of their wealth”, since private wealth had risen sharply in the previous years and the previous tax incentives were considered insufficient to encourage individuals to part with a “significant” portion of their wealth (Report 2003, p. 7428, 7426–7427; Schiesser initiative* - Parliamentary Initiative No. 00.461*). Such a justification was the only tax policy objective expressly stipulated by the legislator; thus, at least one of the goals of the reform was to boost donations (the general goal of the 2006 reform was “the liberalization of the Swiss foundation law in order to boost the establishment of foundations”) (Report 2003, p. 7426; Lideikyte-Huber and Pittavino 2022).

After the reform at the federal level, cantonal law modifications were introduced; in the canton of Geneva, the 5% deduction threshold for taxable net individual income (Personal Income Tax Act – V) increased to 20% in 2010 (Personal Income Tax Act of September 27, 2009, FR: Loi sur l'imposition des personnes physiques—27 September 2009; LIPP; D 3 08, in force: 01.01.2010) and the deduction threshold for corporations increased from 10 to 20% (Corporate Income Tax Act of September 23, 1994). Table 1 summarizes the timeline with the two deduction ceilings (5% and 20%) for the two reforms regarding charitable deductions, which were computed based on the intermediary net income in the Geneva cantonal individual income tax laws. It is important to note that both reforms from 2001 and 2009, as shown in Table 1, are related to income tax incentives. So far, no dedicated reforms to wealth tax incentives are available.

**Table 1:** Timeline with the two deduction ceilings (5% and 20%) for the two reforms regarding charitable deductions, computed on the intermediary net income, in the Geneva cantonal individual income tax laws

	2001	2002	2003	2004	2005	2006	2007	2008	2009	2010	2011
Cantonal law (GE)	5%								20%		
	1st reform								2nd reform		

One of the aims of this study is to address the gap in exploring the potential for introducing a wealth tax incentive, as evidenced by findings on the behavior of regular donors.

## Data and methods

### Data description

Our analysis is based on information from taxpayer returns over 11 years from 2001 to 2011 that the Tax Administration of the Canton of Geneva (TACG) provided to us confidentially for this study (Hoboken 2021). The selected variables provide information on the entire population of taxpayers in the Canton of Geneva (approximately 250,000 households). A different data set was provided for each year under study, 11 in total. Each data set comprised the same nine variables; a full description of them is provided in Lideikyte-Huber and Pittavino (2022) and Lideikyte-Huber, Pittavino, and Peter (2021); the ones specifically used in the present study are described and listed below with their original name provided in brackets. For this study, two new variables, “year” and “freqded”, were specifically created to enable a more detailed longitudinal analysis. These variables facilitate the examination of the study year and the computation of donation frequency of over time, following panel data literature on charitable giving, econometrics and time-series forecasting (Barrett 1991, Baltagi and Pesaran 2007, Baltagi 2013, Hyndmann and Athanopoulos 2021). The new variable “freqded” is specifically designed to track regular donors over time and explore the continuity of their donations. Meanwhile, the variable “year” is also essential to this study, playing a crucial role in analyzing future donation patterns and donor behavior over the next 10 years, providing a more in-depth longitudinal analysis for both the current and future years of study. 

A merging of the 11 different data sets, with the elimination of double IDs, if any, was performed to create the appropriate unique dataset:

- “Coded ID” (“identifiant”): a coded ID for each taxpayer. This variable allows us to follow the same taxpayer over time. The same coded ID is used for any given taxpayer for each fiscal year. As Switzerland has a joint filing system, married couples are considered and treated the same as a single non-married individual, and they have only one coded ID (in this paper, any deducting taxpayer, couple, or individual is referred to as “deductor” or “deducter” interchangeably).
  - “Year of birth” (“annee_de_naissance”): the year of birth of a taxpayer (which is either an individual or a household, depending on marital status). For married couples, it is the year of birth of the “principal” taxpayer, usually the man.
  - “Income_bracket” (“bareme_revenu”): the binary (0/1) indication of a possible “splitting” of income tax rate in the tax income computation, showing if a taxpayer is a couple (1) and not a single individual (0).
  - “Global net taxable income” (“revenu_net_imposable_taux”): the net taxable income (after all deductions) applied to set the tax rate; this includes any and all foreign income.
  - “Gross wealth” (“fortune_brute”): global gross wealth of the taxpayer.
  - “Deductions for donations” (“versements_benevoles”): the amount of deduction (if any) for charitable giving, representing the entire annual amount of the deducted donations (in case it is less than the deductible threshold) or capped amount of annual donations, if exceeding the deductible threshold.
  - “Intermediary net income for deductible donations” (“Sous_total_ded_dons”): this variable serves as a key reference point for calculating deductions that are under, equal to, or more than the legal threshold (10% or 20%, depending on the year). It could only be digitally extracted from the databases of the Tax Administration of the Canton of Geneva (TACG) for the tax years 2010 and 2011. For the previous years, it was determined by internal calculations performed by the TACG, based on the elements of the tax base that are included in its definition (information provided by TAGC).
  - “Year under study” (“year”): this new variable has been generated for the purpose of this study to keep track of the evolution of the collected data by year. It indicates the 11 years under consideration for this study, from 2001 to 2011.

“Frequency of deductions” (“freqded”): innovative additional variable created to count the frequency with which a given ID repeated the charitable deductions over the study period. 

This data was selected for taxpayers residing in the Canton of Geneva as well as for taxpayers residing in another Swiss canton or abroad, however still taxed in Geneva. The information above does not allow us to distinguish between these different categories of taxpayers. In addition, as from the 2009 tax year, taxpayers who are usually taxed at source (“*impots à la source”*, a dedicated taxation practice for newcomers in Switzerland) can fill in a tax return, if they meet certain conditions, and are then treated as resident taxpayers (“*quasi-residents*”). There were approximately 2,000 of these taxpayers in 2009, 4,000 in 2010, and 5,600 in 2011. The variables provided by TACG do not allow us to identify quasi-resident taxpayers (*Loi sur l'imposition des personnes physiques (LIPP-V)*).

As reported in Lideikyte Huber, Pittavino, and Peter 2021, the total number of taxpayers in the canton of Geneva saw a steady increase, from 234,117 in 2001 to 266,336 in 2011. The share of the taxpayers deducting charitable donations more than doubled, growing from 8.3% in 2001 to 19.3% in 2011, with a steep increase in 2005 (deducting taxpayers reaching 16.3%). Concerning the general pattern of deductions during the studied period, the total amount of yearly charitable deductions increased significantly, from CHF 29,133,697 in 2001 to CHF 72,741,235 in 2011 (amounts not adjusted for inflation) which is due to the rise in population; a substantial increase of 48% is recorded in 2009.

In the present analysis, we use the terminology of “deductors” or “deducters” (interchangeably) to indicate the taxpayers who contributed to charitable donations and used a tax incentive (deduction) in relation to their donation, since we want to investigate this specific subset of the taxpayer population.

### Data subset and description by frequency of deducters and deduction ceiling

The last described variable “freqded” has been generated to allow innovative data analysis by highlighting the frequency of donations from the deducters over the 11 years under study. 

Table 2 indicates the frequency of deductions by each deducter, the total number of deducters within each frequency value, and the resulting percentage of deducters. We observe that 29.4% of deductors (corresponding to a total of 30,319 deductors) donated only once. The remaining 70.6% of deductors donated more than once, showing knowledge of the tax incentives for charitable deductions, a related interest for this fiscal advantage, and the start of repeated behavior within their donations. With this targeted analysis, it was possible to identify a specific group of deductors who donated over the entire period of 11 years. This subgroup corresponds to 5,948 taxpayers, who represent 2.54% of the starting Geneva taxpayer population from 2001 to those people who gave consistently from 2001 to 2011. This subgroup of deducters will be called *deducters11* from now on and it will be compared to another subgroup of deducters identified in Lideikyte-Huber and Pittavino 2022, who are more interested in targeting the ceiling of deductions, referred to as ‘deducter subset’.

**Table 2:** Frequency of deductions, total number, and percentage of deducters

Frequency of deductions	Total number	Percentage
1	30,319	29.4
2	15,597	15.1
3	10,994	10.7
4	8530	8.3
5	7003	6.8
6	5739	5.5
7	5378	5.2
8	4677	4.5
9	4467	4.3
10	4490	4.4
11	5948	5.8

**Fig. 1:**
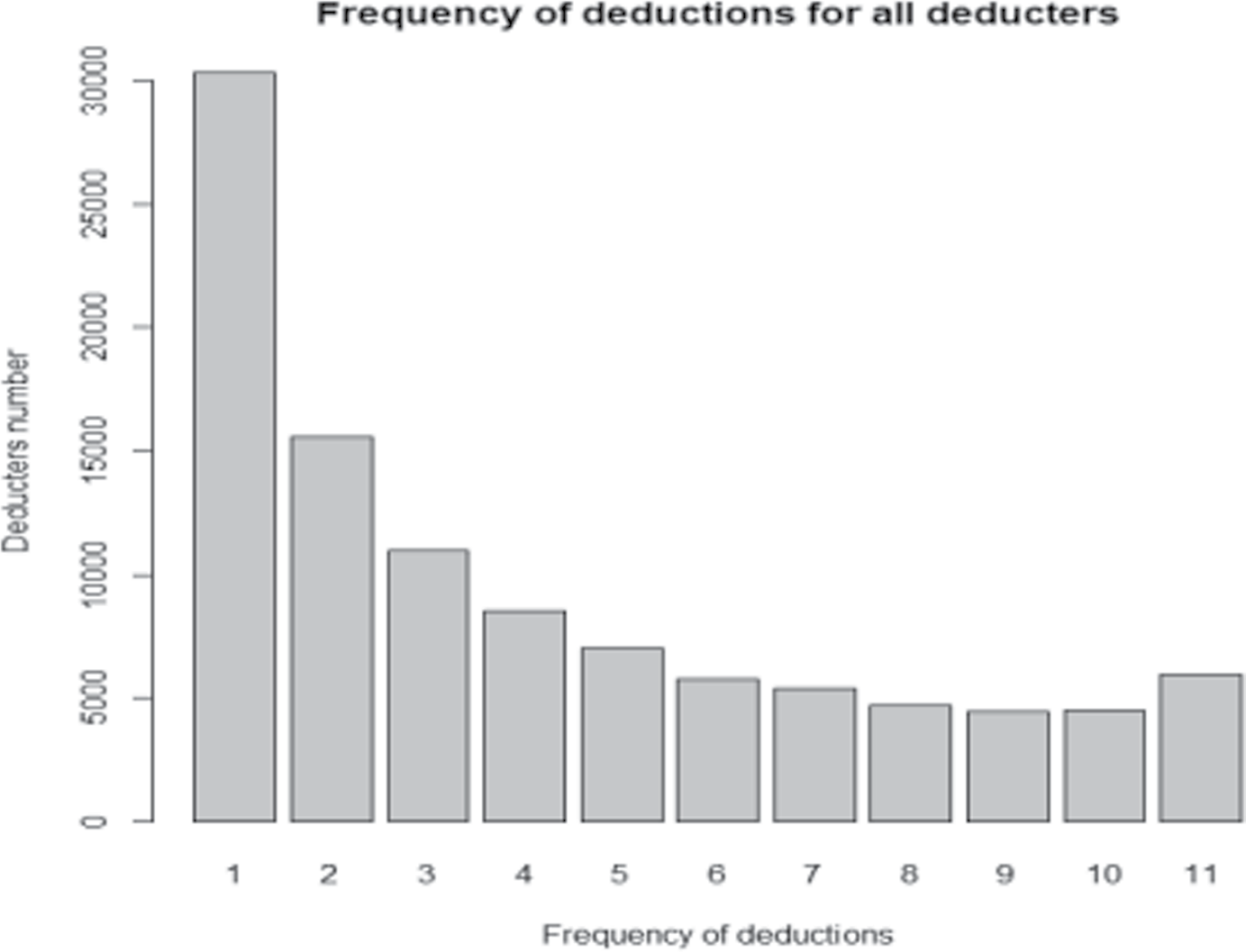
Bar graph representing the frequency of deductions by each deducter over a total of 11 years

The figures related to the frequency of deductions have also been represented in the bar graph in Fig. 1, where for each frequency, the total number of deducters is shown. This graph shows the declining pattern of the frequency of deductions for all deducters, the majority presenting as people who deducted once, as well as those who repeated the process over time more than once, which reflects a repeated behavior in the deductors and an awareness of the tax incentives by the entire taxpayer population. However, this declining pattern plateaus considerably. It is interesting to observe that the number of donors who give very regularly, from 5 to 11 years during the studied period, is very similar. For instance, the number of donors who give once every two years and once a year is nearly the same.

**Fig. 2:**
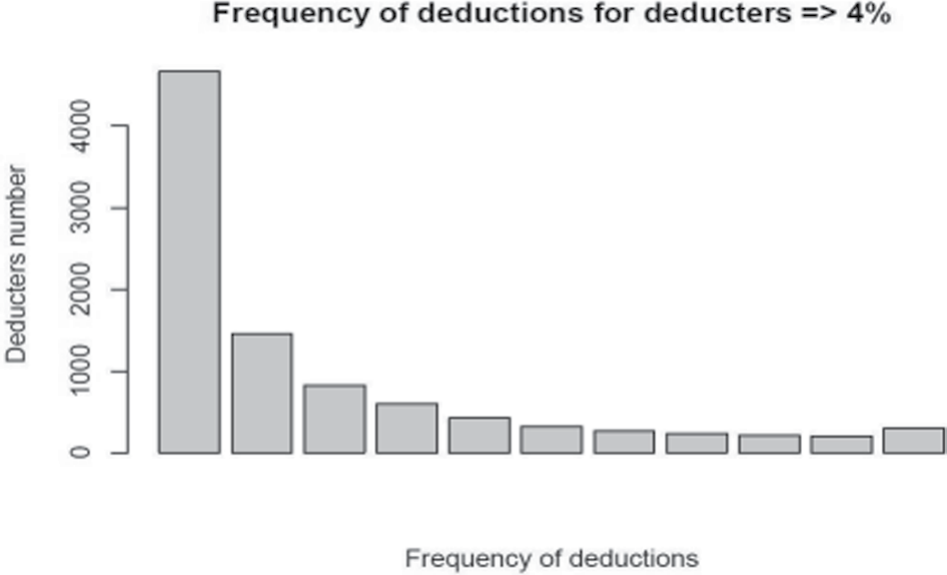
Bar graph representing the frequency of deductions by each deducter, who donated the legal ceiling of 4% and more, over the 11 years under study

In Table 3, the figures for the frequency of deductions for deducters interested in reaching the ceiling of deductions of 5% of their taxable income and more are reported. Similarly to Table 2, the first three columns report the frequency of deductions, the total number of taxpayers within that frequency, and the corresponding percentage relative to the overall population. In addition, compared to Table 2, an extra column is included to show the percentage of deducters within this subgroup who reached the legal deductible threshold (*deducters11*). In Lideikyte-Huber and Pittavino 2022, the 4% threshold was chosen to observe taxpayers who might be targeting the legal 5% deductible threshold, to which the tax incentive was limited during most of the study period. The reason for lowering the threshold in our work was the fact that it is not easy for taxpayers to predict the exact taxable income for the year in progress—it depends on several factors, such as which deductions will be accepted by the tax authorities upon filing tax returns. Thus, we estimated that the taxpayers who wanted to reach the deductible ceiling but did not exceed it might have cautiously estimated their maximum number of deductions available and therefore did not fully reach the official ceiling of 5% (because everything exceeding that is non-deductable). In the third column of Table 3, 49% of all deductors who reached this legal ceiling donated only once. Moreover, of the 3.1% who regularly donated over the 11 years under study, they represent only 5% of the total of constant deductors (*deducters11*). These first results highlight that taxpayers who reached the legal ceiling for deduction were not constant deducters. Half of the taxpayers who reached the above ceiling donated only once, as also shown in Fig. 2. From now on we will call *deducters11-ceiling* the constant deducters who reached or exceeded the donation ceiling for tax incentives.

**Table 3:** Frequency of deductions, total number, overall percentage and percentage over regular deducters (deducters11) for taxpayers who donated amounts reaching or exceeding the ceiling of 5% (deducters11-ceiling)

Frequency of deductions	Total number	Percentage	Percentage over *deducters11*
1	4667	49.0	15.4
2	1459	15.3	9.4
3	826	8.7	7.5
4	604	6.4	7.1
5	424	4.5	6.1
6	323	3.4	5.6
7	265	2.8	4.9
8	235	2.5	5.0
9	213	2.2	4.8
10	202	2.1	4.5
11	297	3.1	5.0

The third variable described above (“splitting”) and presented in our dataset shows whether the taxpayer’s household is entitled to specific rates that apply to spouses, registered partners (same-sex couples), or taxpayers who live in the same household as their minor or adult children, or a close relative who is a family dependent. By analyzing all the deducters, the *deducters11,* and *deductes11-ceiling*, this variable always shows an equally distributed population between these characteristics, even with a prevalence of joint filling. This indicates that the regularity of deductions is typical of family households (married couple, a person with dependents, etc.) rather than single taxpayers, as revealed by Lideikyte-Huber and Pittavino (2022), while looking at the population’s subset features.

### Descriptive statistics

The descriptive statistics for the dataset of *deducters11* and *deducters11-ceiling* with the main features are summarized in Table 4 and Table 5, respectively. If we compare Table 4 to Table 5, which is a special case of Table 1 in Lideikyte-Huber and Pittavino (2022) for the subgroup of people donating who were interested in reaching the ceiling, we can find overall higher values.

**Table 4:** Summary statistics of the *deducters11* constant donors who deducted for the entire 11 years of the time period under study, without taking into account the deduction ceiling (n = 5948)

Year	Total deductions	Average deduction	Median deduction	Average net global income	Median net global income	Average gross-wealth	Median gross wealth	Average year of birth	Median year of birth	Single	Married^1^, or single with dependents^2^
2001	15,388,211	2587	736	211,194	88,552	2,126,201	431,338	1944	1943	2439	3509
2002	15,987,318	2687	860	187,188	90,846	2,087,835	454,395	1944	1943	2417	3531
2003	14,267,464	2399	950	178,421	90,233	2,230,951	483,034	1944	1943	2411	3537
2004	16,375,487	2753	1050	183,617	89,732	2,339,291	505,483	1944	1943	2416	3532
2005	18,466,780	3105	1150	189,970	89,314	2,582,534	548,907	1944	1943	2387	3561
2006	17,037,744	2864	1100	207,383	90,328	2,820,589	575,364	1944	1943	2383	3565
2007	18,895,196	3177	1176	241,464	92,284	3,085,099	588,272	1944	1943	2392	3556
2008	20,963,104	3524	1′215	236,625	93,282	2,870,672	591,562	1944	1943	2408	3540
2009	34,477,153	5796	1′270	192,308	92,600	3,112,107	624,118	1944	1943	2422	3526
2010	32,331,721	5436	1′310	186,050	82,861	3,277,245	639,482	1944	1943	2425	3523
2011	23,467,958	3946	1315	179,976	82,219	3,296,511	647,618	1944	1943	2441	3507

^1^The civil status « married» also includes registered same-sex partners under Swiss law

^2^A single, widowed, divorced, separated person and those living with minor children, or children between the ages of 18 and 25 who are studying or doing an apprenticeship, or a relative for whom the taxpayer is essentially responsible financially

**Table 5:** Summary statistics of the *deducters11-ceiling* constant donors who deducted for the entire 11 years of the time period under study, without taking into account the deduction ceiling (n = 297)

Year	Total deductions	Average deduction	Median deduction	Average net global income	Median net global income	Average gross-wealth	Median gross wealth	Average year of birth	Median year of birth	Single	Married^1^, or single with dependents^2^
2001	2,461,132	8287	3977	156,574	74,863	2,820,897	411,509	1941	1939	139	158
2002	2,302,032	7751	3933	145,905	74,702	2,642,858	403,154	1941	1939	140	157
2003	2,170,172	7307	3924	137,537	74,099	2,689,108	417,519	1941	1939	140	157
2004	2,238,344	7536	3764	141,340	69,909	2,728,003	429,240	1941	1939	141	156
2005	2,261,060	7613	3826	143,281	71,719	2,977,176	472,066	1941	1939	141	156
2006	2,481,737	8356	3851	157,149	70,738	3,057,097	499,964	1941	1939	141	156
2007	2,487,059	8374	3909	157,296	72,951	3,920,514	496,051	1941	1939	140	157
2008	2,751,581	9265	3961	174,123	73,844	3,474,699	468,818	1941	1939	142	155
2009	4,465,830	15,036	7658	142,201	69,127	3,566,142	511,085	1941	1939	145	152
2010	4,655,780	15,676	8060	121,683	58,710	3,557,364	516,158	1941	1939	144	153
2011	6,113,253	20,583	7300	123,151	60,539	3,503,996	515,717	1941	1939	145	152

^1^The civil status « married» also includes registered same-sex partners under Swiss law

^2^A single, widowed, divorced, separated person and those living with minor children, or children between the ages of 18 and 25 who are studying or doing an apprenticeship, or a relative for whom the taxpayer is essentially responsible financially

In the subgroup dataset *deducters11*, the total amount of deductions is higher, and the median and mean age of taxpayers is higher including younger people and those in taxpayer households (married or single-sex registered partners, taxpayers with dependents, etc.). The median and mean values for the global income and gross wealth are also higher than the subgroup of deducters *deducters11-ceiling*. The only lower values are the median and the mean deductions, since they are not necessarily interested in reaching the ceiling, the *deducters11* donated, on average, less than the *deducters11-ceiling*.

**Fig. 3:**
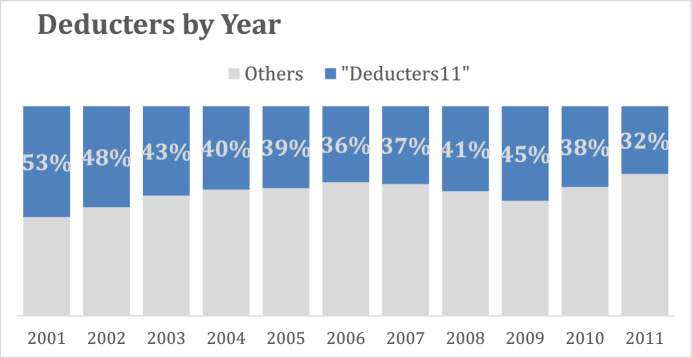
The total amount of deductions each year for all the subgroups of constant donors: *Deducters11*, represented by the blue line, versus all the other donors who did not deduct for the entire period

The percentage of the number of deductions for 11 years from *deducters11*, in blue, compared to the annual total amount of deductions, the entire bar, is represented in Fig. 3. This group of regular taxpayers contributed to almost half of the total deductions at the beginning of the study period. The relative percentage of *deducters11* compared to the entire deducter/donor population decreased over time, which is explained by the fact that, in absolute terms, the number of deducters increased during the study period (Lideikyte-Huber and Pittavino 2022).

### Methods

Two different classes of statistical methods have been used with two different aims. Firstly, regression methods (linear and linear mixed models) with and without interaction have been applied. In particular, linear bivariable models applied to two subgroups of deducters: *deducters11* and *deducters11-ceiling* to identify the main significant variables driving the charitable deductions in regular deducters, who did and did not reach the ceiling threshold. Secondly, forecasting methods applied to the amount of deductions and the amount of donors, to project the predicted figures in the next upcoming 10 years: 2012–2021.

The entire data set, without any subsetting, was analyzed in detail in Lideikyte-Huber and Pittavino (2022). For the Exploratory Data Analysis (EDA) we proceeded to focus on the new subset of datasets *deducters11* and *deducters11-ceiling*. The main summary statistics (e.g., mean, SD, min, max, median) have been checked and computed. Since some of the variables represent almost the same quantity (i.e., “global net taxable income” (“revenu_net_imposable_taux”), “intermediary net income for deductible donations” (“Sous_total_ded_dons”)), they share the same part of the variance to describe the response which resulted in a very high multicollinearity between some pairs of variables. The variance inflation factor (VIF) (Faraway 2002) has been calculated to measure the amount of variance presented by each one of them for the resulting model. This quantity was computed to select the optimal set of variables for our analysis. This is an indication of the presence of multicollinearity. Two variables, X1: “global net taxable income” and X2: “gross wealth” resulted in an overall Mean for the VIF of 2.75.

### Bivariable linear regression analysis with and without interactions

The first method used to analyze the data for the frequency of deductions of *deducters11* and *deducters11-ceiling* was a bi-variable linear regression analysis between income (X1) and wealth (X2), because of the VIF check, with and without interaction (Faraway 2004 and 2016; Pittavino et al. 2017a and 2017b).

For the deducter*s11* first sub-dataset, with ${Y}_{i}$ = deductions for donations, i = 1, …, 5948, we have:

1 $${Y}_{i}={\beta }_{0}+{\beta }_{Inc}{X}_{i1}+{\beta }_{Wth}{X}_{i2}+{\beta }_{Int}{X}_{i1}{X}_{i2}+{\varepsilon }_{i}$$

Model 1), with interaction.

2 $${Y}_{i}={\beta }_{0}+{\beta }_{Inc}{X}_{i1}+{\beta }_{Wth}{X}_{i2}+{\varepsilon }_{i}$$

Model 2), without interaction.

With ${\upvarepsilon }_{\mathrm{i}} \sim \text{ N}\left(0, {\upsigma }^{2}\right)$, independent and identically distributed (iid).

${{\beta }_{Inc}, \beta }_{Wth} \mathrm{and} {\beta }_{Int}$ are the regression coefficients for income, wealth, and their interaction.

For the *deductors11-ceiling* second sub-dataset, we have:

${Y}_{j}$= deductions for donations, j = 1, …, 297

3 $${Y}_{j}={\beta }_{0}+{\beta }_{Inc}{X}_{jInc}+{\beta }_{Wth}{X}_{jWth}+{\beta }_{Int}{X}_{j1}{X}_{j2}+{\varepsilon }_{j}$$

Model 3), with interaction.

4 $${Y}_{j}={\beta }_{0}+{\beta }_{Inc}{X}_{jInc}+{\beta }_{2Wth}{X}_{jWth}+{\varepsilon }_{j}$$

Model 4), without interaction.

With ε_j_ ~ N(0, σ^2^), independent and identically distributed (iid).

Given the panel nature of our data set and the repetition over time for the frequency of deductions, bi-variable linear regression models with random effect: *Linear Mixed-Effects Models* (LME; Pinheiro and Bates 2000), with and without interaction, have also been performed using the *lme4 R package* (Bates et al. 2014), to incorporate better the subject variability, with and without interaction for income and wealth. LME have been fitted to check if there was subject variability, that was impacting the related estimates.

Two types of LME models have been implemented, with a random intercept: ${b}_{0i},\text{ modelled as Gaussian distribution with mean }0\text{ and variance} {\sigma }_{b}^{2}:{b}_{0i}\sim N\left(0, {\sigma }_{b}^{2}\right)$ for each subject *i* and with random slope ${b}_{1i}$, modelled as a Gaussian distribution with mean 0 and variance ${\sigma }_{1}^{2}:{b}_{1i}\sim N\left(0, {\sigma }_{1}^{2}\right)$ for each year (timepoint) of deductions.

We also performed *robust regression analysis* based on the Least Median of Squares (LMS) method: $\underset{{\beta }_{0}{\beta }_{1}}{\mathrm{min}}\mathrm{med}$ ${\varepsilon }_{i}^{2}$, the median of the errors is minimized instead of the sum of the square errors. There is no analytic expression for the LMS, but search algorithms and the class of *M-estimators* is used: $\underset{{\beta }_{0}{\beta }_{1}}{\mathrm{min}}{\sum }_{i=1}^{n}\rho \left(\frac{{\varepsilon }_{i}}{\sigma }\right)$, Huber and Ronchetti 2009. An M-estimator is robust if the function ρ limits the extremes values $\frac{{\varepsilon }_{i}}{\sigma }.$ The function Tukey’s bisquare (or biweight), satisfying the previous criteria, is used:

5 $${\rho }_{c}\left(\frac{{\varepsilon }_{i}}{\sigma }\right)=\left\{\begin{array}{c}\frac{6}{c}\left[{\left(\frac{{\varepsilon }_{i}}{c\sigma }\right)}^{6}-3{\left(\frac{{\varepsilon }_{i}}{c\sigma }\right)}^{2}+3 {\left(\frac{{\varepsilon }_{i}}{c\sigma }\right)}^{2}\right] if \left|\frac{{\varepsilon }_{i}}{\sigma }\right| < c,\\ \frac{6}{c} if \left|\frac{{\varepsilon }_{i}}{\sigma }\right| \ge c,\end{array}\right.$$

The robust regression is implemented using the *robust R *package (Wang et al. 2023), with and without interaction, for both the two datasets *deducters11* and *deducters11-ceiling* to further check our findings.

### b. Time-series forecasting methods with applications in philanthropy

The second class of methods used to make predictions of the current values for the next upcoming 10 years were the ETS (Error-Trend-Seasonality) and the ARIMA Models (Hyndman and Athanopoulos 2021). Four different types of ETS models (from Simple Exponential Smoothing (SES) to Holt’s Models), considering different trend effects (i.e. additive, additive with damped and multiplicative) since the presence of seasonality was not detected, have been fitted to compute the amount of donations for the upcoming 10 years (2012–2022). Five different error metrics (i.e. AIC, AICc, BIC, RMSE and MAPE) have been calculated and the smallest ones have been implied to select the best model for the forecast.

The ETS models (Pittavino and Lideikyte-Huber 2024, Pittavino 2025) represent an evolution of the standard Moving Average estimation technique for the trend component of the time series with the inclusion of specific weights which will base and update the forecast, not only on the last observations but also taking into account all the time series history; from this the weighted average origin. The ETS models are used when the whole time-series, representing the time span of the philanthropic data is available, and the model performance is based on the time already collected and the future time window to forecast. This type of model takes its name from the single part of the time series they are predicting.

Generally, the philanthropic time-series data is decomposed into three habitual components (E, T, S):

- Error (E) indicates the difference between the true and the estimated values.
- Trend (T) represents the increasing or decreasing pattern of the data.
- Seasonality (S) shows the presence of higher or slower changes in data behavior.

As the name suggests, this methodology involves estimating each time-series component mentioned above individually, using either an additive or multiplicative decomposition, resulting in a total of 30 possible model combinations: 15 combinations from including an additive error, and another 15 from including a multiplicative error. The Additivity (A) is chosen when the changes happen in time with a linear and slight variation, while the Multiplicativity (M) is chosen when the changes occur in time with an exponential and considerable variation.

Since philanthropic data generally lack seasonal effects, only the trend component was modeled. Accordingly, four ETS models (from SES to Holt’s), with three types of trends (additive, additive damped, and multiplicative), were fitted for comparison.In the *R* software, those types of models are implemented using the *ets* function from the *fpp2* and *fpp3* packages, which are essential tools to work with this type of model.

The last class of models we will describe is the AutoRegressive Integrated Moving Average (ARIMA) model, which extends the AutoRegressive Moving Average (ARMA) model (Hyndman and Athanopoulos 2021, Pittavino and Lideikyte-Huber 2024, Pittavino 2025). ARIMA models differ from regression models in that they do not explicitly define the relationship between the outcome variable *y*_*t*_ (expressed in terms of time) and the covariates *x*_*t*_. Instead, they use linear models to express the relationship between *y*_*t*_ and its own past values *y*_*t−1*_.

The full and compact formulation of the ARIMA model can be found in the mathematical Eq. (6) below:

6 $$ y^{\prime}_{t} = c + \phi_{p} y^{\prime}_{t - 1} + \ldots + \phi_{p} y^{\prime}_{t - p} + \theta_{1} \varepsilon_{t - 1} + \cdots + \theta_{q} \varepsilon_{t - q} + \varepsilon_{t} $$

The ϕ parameters represent the AutoRegressive (AR) model part, where the autoregression states that the current observation y_t_ can be predicted by a linear combination of the past observations y_t-p_. At the same time the θ parameters represent the Moving Average (MA) model part, where the current observation y_t_ can be predicted by a linear combination of past forecast errors ${\varepsilon }_{t-q}$.The software R implements them through the *Arima* or *AutoArima* function present in the package *fpp2*.

## Empirical results

### Outcome from the regression models for 11-years of deductions: regular donors—deducters11

In this section, the results of the regression models are presented and discussed.

From Table 6 we can observe how the interaction between income and wealth is significant, even if smaller (i.e. all 11 years: βInt = *1.9* × *10–11*) on a global level than all the regular deductors, indicating that higher income and wealth positively influence charitable deductions and vice versa. The income variable per se has a negative effect on deduction, showing that wealth is the main variable influencing the deductions for regular deductors. These aggregated results for the entire 11-year time period have been granulated by each year to better understand the dynamics: for 5 years out of 11 (2001, 2005, 2008, 2009, 2010) the regression coefficients of the income were negative (i.e. 2001: βInc = *− 2.2* × *10–3*) showing a negative relationship to the response variable charitable giving and highlighting how wealth has a positive effect on the outcome.

**Table 6:** Table showing the β estimates and p-values for the variables: Net Income for deductible donations (Inc), Gross Wealth (Wth) and their interaction term (Int), along with the adjusted R^2^ (R^2^Adj), from standard bivariable linear regression model (OLS)-with and without interaction-fitted for the subset of *deducters11*

	Linear models with interaction							Linear models without interaction				
Deducters11	βInc	pInc	βWth	pWth	βInt	pInt	R2Adj	βInc	pInc	βWth	pWth	R2Adj
All years	***− 4.3* × *10–3***	** < 0.05**	**9.2 × 10–4**	** < 0.05**	**1.9 × 10–11**	** < 0.05**	**0.31**	4.4 × 10–3	< 0.05	9.7 × 10–4	< 0.05	0.30
All years^1^	***− ****8.0* × *10–3*	< 0.05	1.3 × 10–3	< 0.05	1.4 × 10–11	< 0.05	0.31	− 1.4 × 10–3	< 0.05	1.4 × 10–3	< 0.05	0.30
All years^2^	*− 8.1* × *10–3*	< 0.05	1.3 × 10–3	< 0.05	1.4 × 10–11	< 0.05	0.31	− 2.5 × 10–3	< 0.05	1.5 × 10–3	< 0.05	0.30
All years^3^	*− 9.9* × *10–4*	< 0.05	1.7 × 10–4	< 0.05	1.2 × 10–10	< 0.05	0.13	− 7.4 × 10–4	< 0.05	1.4 × 10–4	< 0.05	0.35
2001	*− 2.2* × *10–3*	< 0.05	4.7 × 10–4	< 0.05	2.5 × 10–11	< 0.05	0.56	6.5 × 10–3	< 0.05	5.5 × 10–4	< 0.05	0.51
2002	2.8 × 10–3	< 0.05	4.1 × 10–4	< 0.05	2.2 × 10–11	< 0.05	0.62	9.5 × 10–3	< 0.05	5.0 × 10–4	< 0.05	0.61
2003	5.3 × 10–3	< 0.05	3.2 × 10–4	< 0.05	*− 1.5* × *10–12*	< 0.05	0.66	4.7 × 10–3	< 0.05	3.2 × 10–4	< 0.05	0.66
2004	4.4 × 10–3	< 0.05	2.9 × 10–4	< 0.05	1.2 × 10–11	< 0.05	0.69	8.6 × 10–3	< 0.05	3.3 × 10–4	< 0.05	0.68
2005	*− 2.8* × *10–3*	< 0.05	2.7 × 10–4	< 0.05	3.7 × 10–11	< 0.05	0.74	1.1 × 10–2	< 0.05	3.8 × 10–4	< 0.05	0.65
2006	6.8 × 10–3	< 0.05	4.5 × 10–4	< 0.05	*− 9.7* × *10–12*	< 0.05	0.62	2.3 × 10–3	< 0.05	4.2 × 10–4	< 0.05	0.59
2007	2.9 × 10–3	< 0.05	3.5 × 10–4	< 0.05	3.0 × 10–12	< 0.05	0.66	4.5 × 10–3	< 0.05	3.6 × 10–4	< 0.05	0.66
2008	*− 6.9* × *10–3*	< 0.05	7.7 × 10–4	< 0.05	2.3 × 10–11	< 0.05	0.58	4.9 × 10–3	< 0.05	8.2 × 10–4	< 0.05	0.53
2009	*− 5.4* × *10–3*	0.02	7.5 × 10–4	< 0.05	8.3 × 10–11	< 0.05	0.48	3.8 × 10–2	< 0.05	9.8 × 10–4	< 0.05	0.43
2010	*− 1.9* × *10–2*	< 0.05	1.9 × 10–3	< 0.05	3.7 × 10–11	< 0.05	0.40	3.0 × 10–4	0.74	2.0 × 10–3	< 0.05	0.38
2011	1.3 × 10–2	< 0.05	5.3 × 10–4	< 0.05	*− 1.9* × *10–11*	< 0.05	0.23	4.4 × 10–3	< 0.05	4.5 × 10–4	< 0.05	0.21

Results are reported for all years and separately for each year under study. Notes: All coefficients significant at p < 0.05 unless otherwise indicated. R^2^Adj refers to the adjusted R^2^ for OLS and the conditional R^2^ for LME models

^1^LME: Linear Mixed-Effects Model with a random intercept

^2^LME: Linear Mixed-Effects Model with a random slope for years

^3^Robust: Robust regression with M-estimation and 90% efficiency

LME was fitted to check if there was subject variability, but they did not show an increase in variance and/or in the model coefficient, especially in the variance description. Even if the resulting standard error for each coefficient was slightly smaller, they did not give added value to the results obtained with Model 1 and Model 2. This result is the consequence of the homogeneity characteristics of this population as seen in Table 4 and Table 5, which remain consistent all over the years and do not imply a drastic change in their behavior, for which the random effects bring advantages.

Three robust regression models with M-estimation technique, with 90%, 80%, and 70% efficiency and corresponding to the c constants: c = 3.881, 3.116, 2.664, for the function Tukey’s bisquare (5), for an iterative estimation of the coefficients, were also fitted and the results for 90% efficiency are reported in Tables 6 and 7. Also in this case there is a negative regression coefficient for income (βInc = − 9.9 × 10–4) and a positive regression coefficient for wealth (βWth = 1.7 × 10–4) and a statistically significant interaction (βInt = 1.2x-10–10), further confirming the previous findings. Since the corresponding R2Adj coefficients for the robust models were lower than the linear and LME ones, the linear regression results were the ones further analyzed and considered. Given the similar characteristics of the group of people in *deducters11*, not many outliers are present and so the robust technique does not show an advantage over classic ones.

**Table 7:** Table showing with the β estimates and p-values for the variables: Net Income for deductible donations (Inc), Gross Wealth (Wth) and their interaction term (Int), along with the adjusted R^2^ (R^2^Adj), from standard bivariable linear regression model-with and without interaction-fitted for the subset of *deductors11-ceiling*

	Linear models with interaction								Linear models without interaction				
Deducters11	βInc	pInc	βWth	pWth	βInt	pInt	R2Adj	βInc		pInc	βWth	pWth	R2Adj
All years-ceiling	5.1 × 10–3	< 0.05	8.1 × 10–4	< 0.05	1.4 × 10–10	< 0.05	0.76	3.6 × 10–2		< 0.05	3.6 × 10–2	< 0.05	0.73
All years-ceiling^1^	5.1 × 10–3	< 0.05	8.1 × 10–4	< 0.05	1.4 × 10–10	< 0.05	0.76	3.6 × 10–2		< 0.05	1.3 × 10–3	< 0.05	0.73
All years ceiling^2^	5.1 × 10–3	< 0.05	8.1 × 10–4	< 0.05	1.4 × 10–10	< 0.05	0.76	3.6 × 10–2		< 0.05	1.3 × 10–3	< 0.05	0.73
*All years*^*3*^	*5.0* × *10–2*	< *0.05*	*2.0* × *10–9*	*0.49*	*− 6.1* × *10–17*	*0.80*	*1*	*5.0* × *10–2*		< 0.05	*1.7* × *10–9*	*0.17*	*1*
2001-ceiling	5.0 × 10–2	< 0.05	2.5 × 10–6	*0.37*	7.3 × 10–13	*0.19*	0.95	5.0 × 10–2		< 0.05	1.6 × 10–6	*0.55*	*0.97*
2002-ceiling	4.9 × 10–2	< 0.05	7.0 × 10–6	0.30	1.3 × 10–12	0.11	0.98	4.9 × 10–2		< 0.05	8.7 × 10–6	0.19	0.99
2003-ceiling	5.0 × 10–2	< 0.05	8.5 × 10–6	0.04	2.5 × 10–13	0.64	0.98	4.9 × 10–2		< 0.05	9.0 × 10–6	0.02	0.99
2004-ceiling	4.9 × 10–2	< 0.05	5.4 × 10–6	0.08	3.5 × 10–13	0.40	1	5.0 × 10–2		< 0.05	5.9 × 10–6	0.06	1
2005-ceiling	4.9 × 10–2	< 0.05	9.8 × 10–7	0.61	8.5 × 10–13	0.10	1	4.9 × 10–2		< 0.05	1.8 × 10–6	0.33	1
2006-ceiling	4.9 × 10–2	< 0.05	1.6 × 10–6	0.45	2.6 × 10–13	0.47	1	5.0 × 10–2		< 0.05	1.7 × 10–6	0.38	1
2007-ceiling	5.0 × 10–2	< 0.05	2.5 × 10–6	0.39	*− *1.3 × 10–13	0 .45	1	4.9 × 10–2		< 0.05	4.9 × 10–7	0.67	1
2008-ceiling	4.9 × 10–2	< 0.05	2.1 × 10–5	< 0.05	6.9 × 10–13	< 0.05	1	4.9 × 10–2		< 0.05	9.2 × 10–6	0.004	1
2009-ceiling	7.6 × 10–2	< 0.05	1.3 × 10–3	< 0.05	− 9.1 × 10–11	< 0.05	0.99	3.5 × 10–2		< 0.05	1.4 × 10–3	< 0.05	0.99
2010-ceiling	9.8 × 10–2	< 0.05	6.4 × 10–4	< 0.05	− 5.3 × 10–11	< 0.05	0.99	7.3 × 10–2		< 0.05	7.0 × 10–4	< 0.05	0.99
2011-ceiling	1.0 × 10–1	< 0.05	− 1.4 × 10–4	0.04	2.5 × 10–10	< 0.05	0.99	2.3 × 10–1		< 0.05	− 9.3 × 10–4	< 0.05	0.99

Results are reported for all years and separately for each year under study. All coefficients significant at p < 0.05 unless otherwise indicated. R^2^Adj refers to the adjusted R^2^ for OLS and the conditional R^2^ for LME models

^1^LME: Linear Mixed-Effects Model with a random intercept

^2^LME: Linear Mixed-Effects Model with a random slope for the years

^3^Robust: Robust regression with M-estimation and a 90% efficiency

The negative coefficient on income observed in Table 6 was further investigated to ensure robustness and to justify the model specification through interaction and non-linear analyses. As reported in Table SM1 of the Supplementary Material, the inclusion of the income × wealth interaction significantly improves model fit (F = 1352, p < 0.001 in OLS; χ^2^ = 517, p < 0.001 in LME), confirming that wealth moderates the income effect. Conditional on wealth, higher income slightly reduces deductible donations-an effect likely linked to deduction ceilings or alternative giving channels among high-income taxpayers.

Moreover, in our dataset, *donors are already those who claim charitable deductions consistently over time* and thus belong to a relatively wealthier and higher-income segment. Within this restricted group, variation in income is less influential than wealth in explaining donation behavior. Consequently, the negative income coefficient reflects that, for taxpayers with comparable wealth, higher taxable income may reduce the relative propensity to deduct-possibly due to the progressive tax schedule and deduction ceilings tied to net income. This interpretation aligns with previous studies on income effects under bounded deductibility (e.g., Duquette 2018; Fack and Landais 2010).

To test for non-linear effects of income, we estimated additional models including quadratic and log–log transformations of income, as reported in Table SM2. The negative and significant coefficient of the squared income term (β^2^Inc < 0, p < 0.05) reveals diminishing marginal effects of income on charitable deductions. Log–log models confirmed the same trend, showing flattened or mildly negative elasticities at higher income levels.

Overall, these results indicate that *wealth exerts a dominant influence on charitable giving*, while income shows a moderated and non-linear relationship with donation behavior. The final specification-a linear OLS model with the *income* × *wealth* interaction, highlighted in bold in Table 6-achieves both statistical robustness and conceptual clarity.

These results are further supported by the consistent statistical significance of the wealth variable across all years, even after accounting for heteroskedasticity using HC1 robust standard errors (see Sect. 2 and Table SM3–SM4 in the Supplementary Materials). This provides additional empirical support for the robustness of the estimated relationship.

**Fig. 4:**
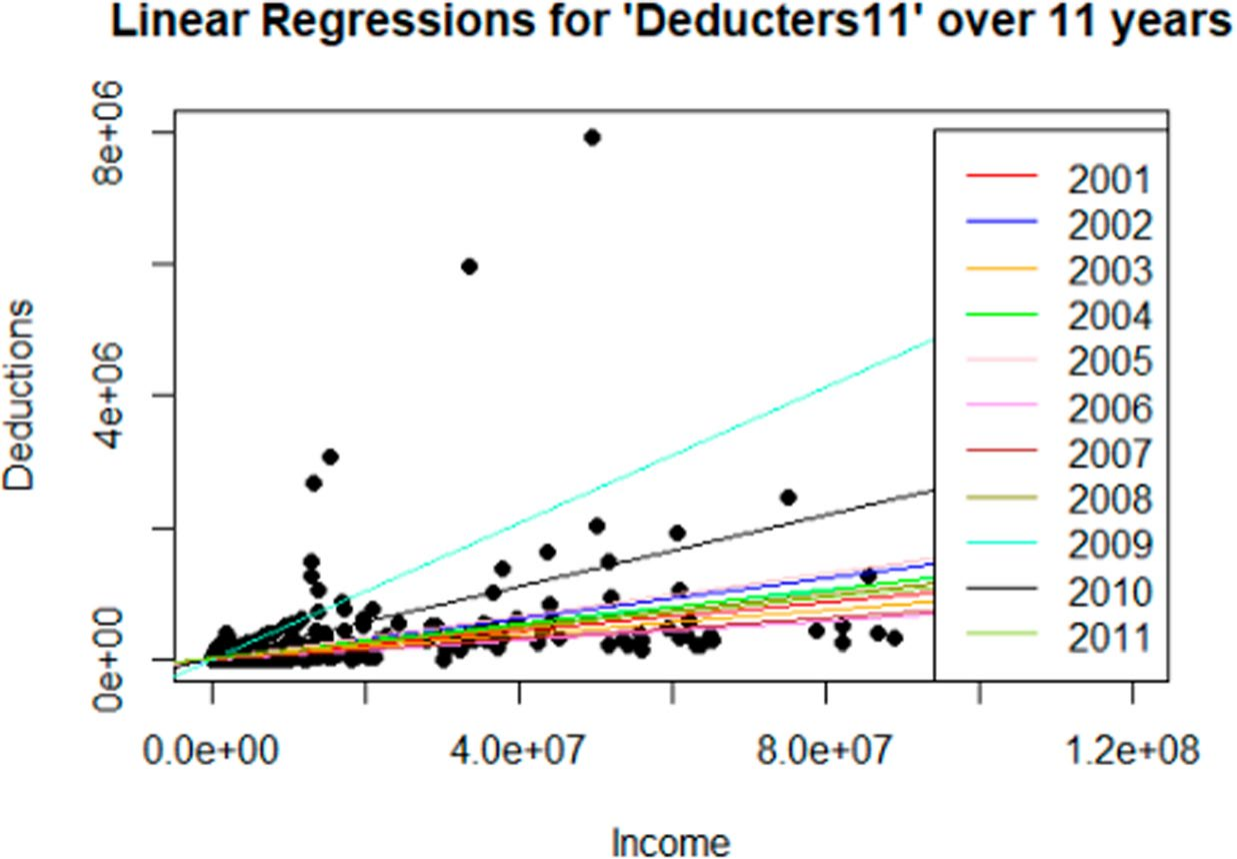
Figure represents the univariate linear regressions with “Deductions” as the outcome variable and “Income” as the explanatory variable, for the entire 11 years of study for the subgroup of consistent *Deducters11*. All 11 regression lines are clearly visible

**Fig. 5:**
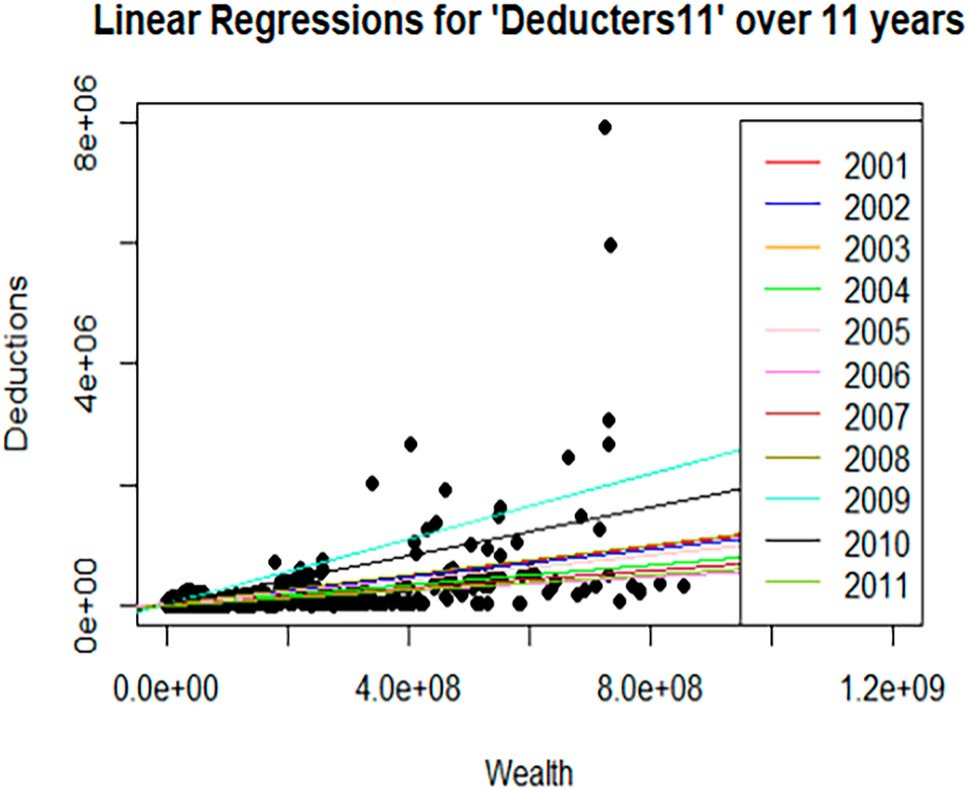
Figure represents the univariate linear regressions with “Deductions” as the outcome variable and “Wealth” as an explanatory variable, for the entire 11 years of study for the subgroup of consistent “*Deducters11*”. In this case, the scale for wealth is 1 million larger, considering the higher range of wealth in comparison to the income scale. All 11 regression lines are clearly visible

As shown in Table 6, wealth remains statistically significant and represents the main driver of donations among taxpayers who consistently claim charitable deductions, without necessarily being motivated by the legal ceiling. This pattern is further illustrated in Figs. 4 and 5, where the regression line with the steepest slope for the *Deducters11* subgroup (light blue) corresponds to the year 2009, when the tax reform came into effect.

### Outcome from the regression models for 11-years of deductions: regular donors reaching the ceiling—deducters11-ceiling

If we observe results from Table 7, referring to the subset *deductors11-ceiling*, the interaction between income and wealth is no longer significant, and only income is a statistically significant variable from the resulting p-value smaller than 0.05. These results confirm the findings from Lideikyte-Huber and Pittavino (2022), where income was the only significant factor for deducters interested in reaching the ceiling. For regular deducters, income has a negative effect on deductions over time, while its interaction with wealth and wealth itself is significant and positive. For the robust regression analysis, similar results as the ones shown in Tables 6 and 7 were found. Moreover, given that there are taxpayers with similar characteristics in the group of regular deducters, there were not so many outliers. We preferred privileges the efficiency of the ordinary least square method rather than the robustness, considering the reduced number of extreme values.

The robust regression showed an advantage on the classical linear for the specific case of *deducters11- ceiling*. Both the variable wealth and its interaction with income were not significant, highlighting that, in the specific subgroup of taxpayers aiming to reach the deduction ceiling, income is the only variable driving donations, as already discussed in Lideikyte-Huber and Pittavino (2022).

Given the very small sample size of 297 regular deductors who reached the deduction threshold (*deductors11-ceiling)*, and the limited set of explanatory variables, the linear models reported in Table 7 yield a near-perfect fit (R^2^Adj = 1).

This result is a direct consequence of the specific construction of this subgroup: it includes only taxpayers who consistently donated at or above the legal deduction ceiling, set at 5% of taxable income before 2009 and increased to 20% after the tax reform.

Because the deduction ceiling is mechanically tied to income and because this subgroup is defined by donors at or above that threshold, the relationship between income and donations is almost deterministic rather than behavioral. In other words, the high R^2^ values reflect the structural definition of the subgroup rather than overfitting or model misspecification. These results should therefore not be interpreted as evidence of behavioral responses in the same way as for the broader donor population. Instead, they confirm the mechanical nature of giving among ceiling-constrained taxpayers (Lideikyte-Huber and Pittavino 2022).

**Fig. 6:**
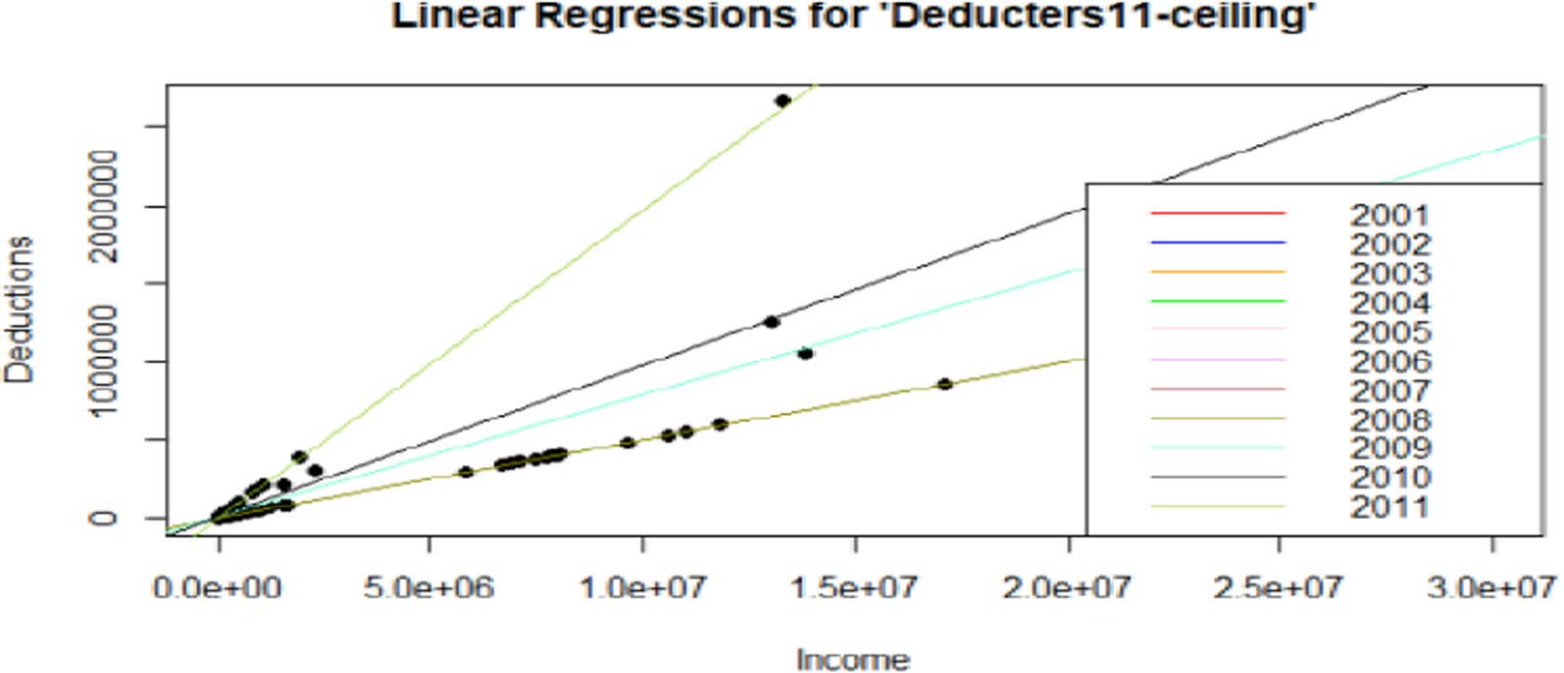
Figure represents the univariate linear regressions with “Deductions” as the outcome variable and “Income” as the explanatory variable, for the entire 11 years of study for the subgroup of consistent “*Deductors11-ceiling*”. For the years 2001 through 2008, the regression lines overlapped with the same slope, and only the models for the last four years are clearly visualized. This indicates the importance of the Income variable for this subset of deducters, interested in reaching the ceiling and consistently donating

**Fig. 7:**
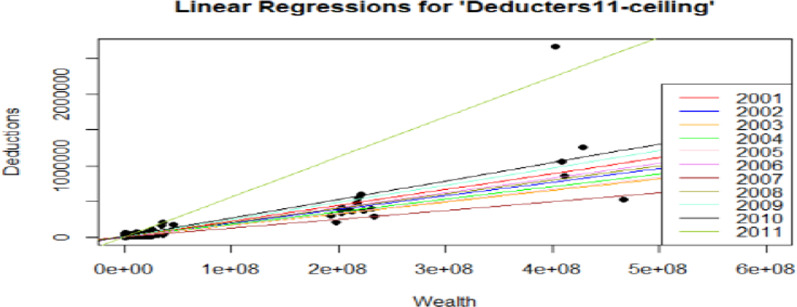
Figure represents the univariate linear regressions with “Deductions” as the outcome variable and “Wealth” as the explanatory variable, for the entire 11 years of study for the subgroup of constant “*Deducters11-ceiling*”. Also, in this case the scale for wealth is larger by 1 million. All the different 11 models are clearly represented, indicating a different slope for each year and how wealth for this particular subset is not a significant variable driving donation

To better explain and illustrate the findings above, the univariate linear regressions over the entire 11 years of study for both the dataset *deducters11* and *deducters11-ceiling*, considering the income and wealth variables as explanatory variables separately, have been represented in Figs. 4, 5, 6, 7. From Figs. 4, 5, 6, 7 it is interesting to observe how the number of deductions diminishes considerably when considering the reduced subset *deducters11-ceiling* of people interested in reaching the ceiling, moving from a maximum of 7,912,630 CHF (approx. 8 million CHF), for *deducters11*, to a maximum of 2,663,594 CHF (approx. 2.5 million), for *deducters11-ceiling*. A similar reduction can be observed in the income range, while the *deducters11* reaches the value of 89,048,109 CHF (approx. 89 million CHF), while the *deductors11- ceiling* reaches the value of 17,106,874 CHF (approx. 17 million CHF). As already shown in the results in Table 7 and as illustrated in Fig. 6 the regression lines for the years from 2001 to 2008 overlapped with the same slope of βInc = 5.0 × 10–2 and only the last one referring to the year 2008, represented in olive green, is visible. Figure 6 shows how the Income variable is clearly statistically representative, explaining the Deductions linked to the legal ceiling. While for the univariate case of the linear regression with Wealth as the explanatory variable, it is possible to see different slopes for each year, indicating that was already reported in Table 7: Wealth is not a statistically significant variable for the taxpayers interested in reaching the deduction ceiling. For Figs. 6 and 7, the regression line with the highest slope for the subset “*Deducters11-ceiling*” represented in olive green, corresponds to the year 2011 and is mainly driven by the biggest donation corresponding to 2,663,594 CHF.

### Outcome from the forecast models for the number of deductions and donors

Five forecasting methods: ETS model with several trend effects have been implemented and compared using error metrics to identify the one that is the most suitable to predict the amounts of deductions and with the best data fit. ARIMA models have also been fitted, however they behaved as SES by always producing the same output, therefore they have been discarded for the donations’ forecast. While they have been incorporated for donors’ forecast.

**Fig. 8:**
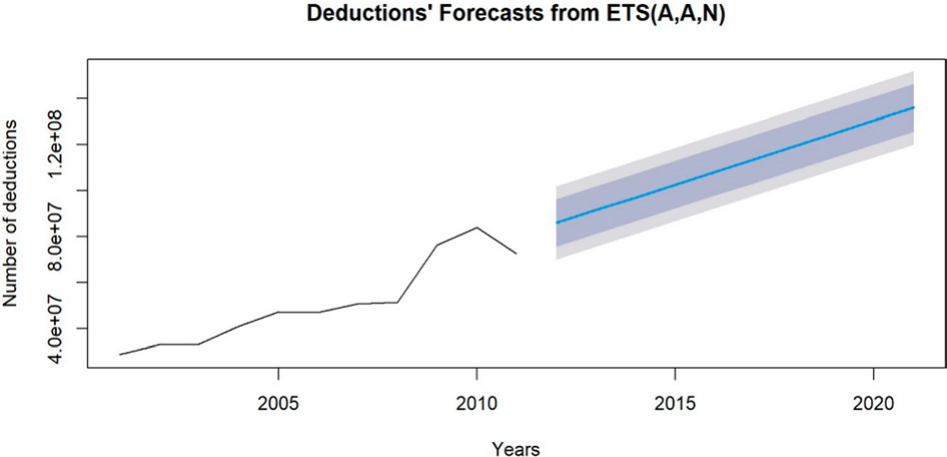
Deductions’ forecasts, over the year 2012–2021. The deductions’ forecast trend is moving from 85′948′989 CHF in 2012 to 135′886′488 CHF in 2021

The best-performing model, with the lowest AICc, and RMSE error metrics term, was model 2) in Table 8, corresponding to a Holt’s model with additive effect and an additive error, capturing the progressively increasing nature of donations over time. However, since multiplicative error was not justified with such an additive decomposition and trend, we decided to choose for the estimation the model with the lowest error metric and respecting the additivity feature of the data. The result of this forecast over the predicted period can be seen in Fig. 8, where the previously mentioned model with the 80% and 95% prediction intervals is shown (Table 9).

**Table 8:** Comparison of the 5 forecasting models (simple exponential smoothing, Holt’s model with and without damped effect for the trend) implemented for donations’ projections and their error metrics

	Errors				
Forecasting models	AIC	AICc	BIC	RMSE	MAPE
SES	389.1287	392.5573	390.3224	11′028′513	16.50398
**ETS(A,A,N)**	**381.3582**	**393.3582**	**383.3477**	**6′458′918**	**10.24257**
ETS(A,Ad,N)	383.3466	404.3466	385.7340	6′455′514	9.902257
ETS(Z,Ad,N)	378.9578	399.9578	381.3451	6′511′752	9.147868
ETS(M,M,N)	383.5166	395.5166	385.5061	8′064′655	12.53688

**Table 9:** Projections of the amount of donations over the following 10 years (2012–2021), resulting from the model ETS(M,Ad,N) with 80% and 95% prediction intervals

Years	Forecast	Lo 80% PI	Hi 80% PI	Lo 95% PI	Hi 95% PI
2012	85′948′989	75′572′672	96′325′305	70′079′782	101′818′195
2013	91′497′600	81,121,283	101′873′916	75′628′393	107′366′807
2014	97′046′211	86,669,894	107′422′528	81′177′003	112′915′418
2015	102′594′822	92,218,504	112′971′140	86′725′613	118′464′031
2016	108′143′433	97,767,113	118′519′752	92′274′222	124′012′644
2017	113′692′044	103,315,723	124′068′365	97′822′830	129′561′258
2018	119′240′655	108,864,331	129′616′979	103′371′437	135′109′873
2019	124′789′266	114,412,939	135′165′593	108′920′043	140′658′489
2020	130′337′877	119,961,546	140′714′208	114′468′648	146′207′106
2021	135′886′488	125,510,152	146′262′825	120′017′251	151′755′725

We did not forecast more than 10 years, because since we had information from the previous 11 years, we did not want to go too far in time to limit the uncertainty.

For the donors’ forecasts, given the data pattern, a multiplicative error for a model with additive damped trend would not be supported, giving similar results as model 3) with an additive error instead. The best model for forecasting the number of donors was an ARIMA specification with two levels of differencing and no autoregressive or moving-average components, as reported in Table 10. The choice was based on the lowest error metrics (AIC, BIC, RMSE) according to the model selection procedure described for the deductions forecasts.

**Table 10:** Comparison of the 5 forecasting models (simple exponential smoothing and Holt’s model with and without damped effect for the trend, several errors and ARIMA model) implemented for donors’ projections and their error metrics

	Errors				
Forecasting models	AIC	AICc	BIC	RMSE	MAPE
*SES*	211.6988	215.1274	212.8925	3466.838	8.264631
*ETS(A,A,N)*	203.8104	215.8104	205.7999	2019.522	5.63453
*ETS(A,Ad,N)*	204.6256	225.6256	207.0130	1913.642	5.161422
*ETS(Z,Ad,N)*	201.7138	213.7138	203.7033	1963.346	3.66038
***ARIMA(0,2,0)***	**162.16**	162.73	**162.36**	1601.337	**2.202275**

**Fig. 9:**
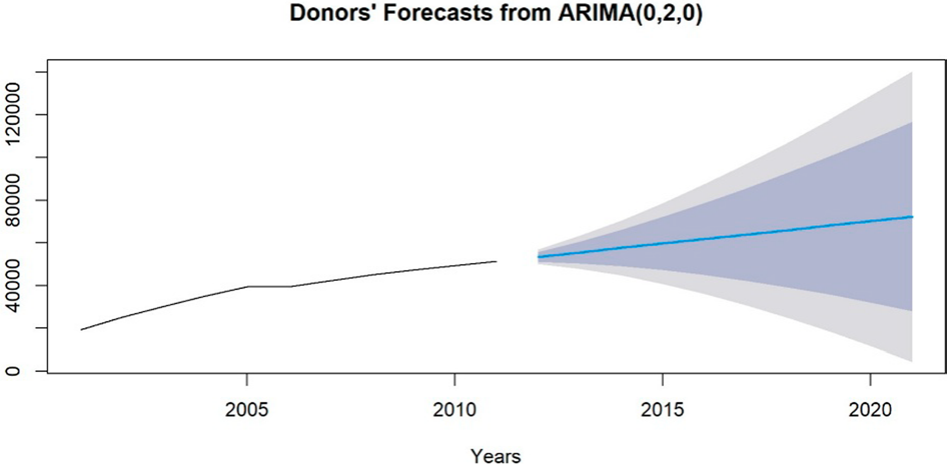
Donors' forecasts over the years 2012–2021, obtained from a twice-differenced ARIMA model with no moving average or autoregressive components. The donors’ forecasts range from 53′485 to 72′349 donors from 2012 to 2021

Furthermore, the results of the stationarity tests that justify the two levels of differencing are presented in Sect. 3 of the Supplementary Material (Table SM5 and SM6) (Fig. 9) (Table 11).

**Table 11:** Projections of the amount of donors over the following 10 years (2012–2021), resulting from the model ARIMA(0,2,0) with 80% and 95% prediction intervals

Years	Forecasts	Lo 80% PI	Hi 80% PI	Lo 95% PI	Hi 95% PI
2012	53′485	51′216.21	55′753.79	50′015.19	56′954.81
2013	55′581	50′507.84	60′654.16	47′822.27	63′339.73
2014	57′677	49′187.97	66′166.03	44′694.15	70′659.85
2015	59′773	47′346.34	72′199.66	40′768.06	78′777.94
2016	61′869	45′043.22	78′694.78	36′136.19	87′601.81
2017	63′965	42′322.14	85′607.86	30′865.11	97′064.89
2018	66′061	39′216.34	92′905.66	25′005.64	107′116.36
2019	68′157	35′752.23	100′561.77	18′598.18	117′715.82
2020	70′253	31′951.45	108′554.55	11′675.84	128′830.16
2021	72′349	27′832.17	116′865.83	4′266.38	140′431.62

**Fig. 10:**
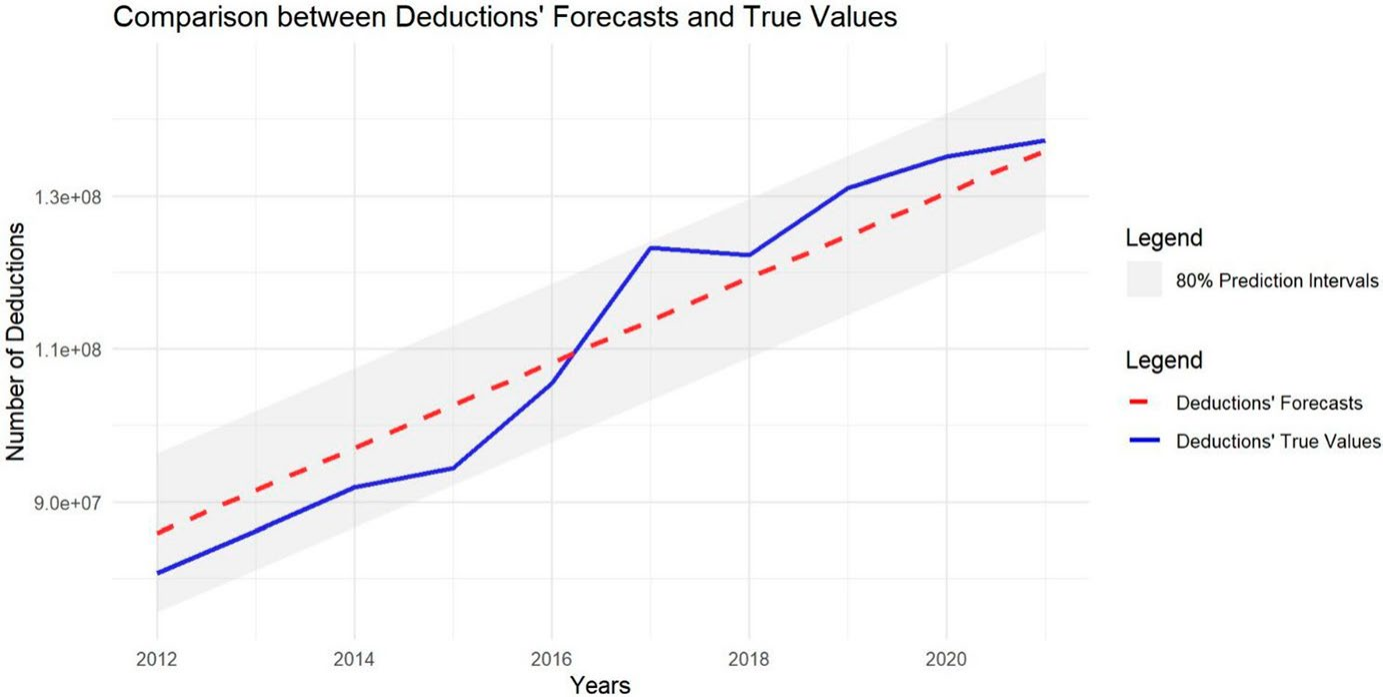
Figure showing the comparison between deductions’ forecasts and true values, with the 80% prediction intervals

**Fig. 11:**
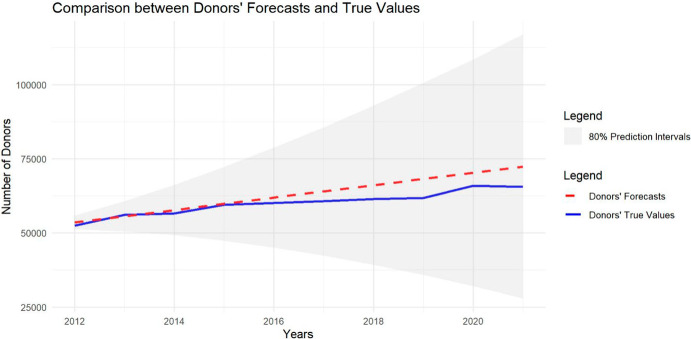
Figure showing the comparison between donors’ forecasts and true values, with the 80% prediction intervals

We were privileged to receive the actual cumulative values for tax deductions and donors from the TACG for the years 2012 to 2021, allowing us to verify the accuracy of our estimates. To do so, we compared our forecasts with the actual values, calculating the Relative Error and RMSE (Root Mean Squared Error) as measures of estimate accuracy. Additionally, we conducted two t-tests on our estimates—one for future deductions and one for future donors—to assess whether the overall estimations were statistically close to the actual values. The results for the number of deductions are presented in Table 12, and Fig. 10 shows the estimated values compared to the actual values, along with the 95% prediction intervals. Similarly, the results for the number of donors are shown in Table 13, with Fig. 11 illustrating the estimated values compared to the actual values and their 80% prediction intervals. In both cases, the estimated deductions and donors fall within the 80% prediction intervals, with p-values of 0.99 and 0.24, respectively. These results suggest that our predictions were statistically close to the actual values, indicating that we provided accurate estimates for the time-series forecasts.

**Table 12:** Relative Errors and RMSE between the Donors’ Forecasts and True Values are reported, together with the overall

Years	Deductions’ Forecasts	True Values	Relative Errors	RMSE	p-value
2012	85′948′989	80′737′888	6.45%	5′211′101	
2013	91′497′600	86′206′400	6.14%	5′291′200	
2014	97′046′211	91′936′496	5.56%	5′109′715	
2015	102′594′822	94′430′131	8.65%	8′164′691	
2016	108′143′433	105′508′401	2.50%	2′635′032	
2017	113′692′044	123′248′199	7.75%	9′556′155	
2018	119′240′655	122′244′114	2.46%	3′003′459	
2019	124′789′266	131′083′788	4.80%	6′294′522	
2020	130′337′877	135′102′899	3.53%	4′765′022	
2021	135′886′488	137′247,977	0.99%	1′361′489	
***Mean***	***110′917′739***	***110′774′629***	**0.13%**	**5′651′443**	**0.99**

**Table 13:** Relative Errors and RMSE between the Donors’ Forecasts and True Values are reported, together with the overall

Years	Donors’ Forecasts	True Values	Relative Error	RMSE	p-value
2012	53′485	52′451	1.97%	1′034	
2013	55′581	56′080	0.89%	499	
2014	57′677	56′550	1.95%	1′127	
2015	59′773	59′457	0.53%	316	
2016	61′869	59′999	3.12%	1′870	
2017	63′965	60′604	5.55%	3′361	
2018	66′061	61′357	7.67%	4′704	
2019	68′157	61′791	10.30%	6′366	
2020	70′253	65′874	6.65%	4′379	
2021	72′349	65′574	10.33%	6′775	
***Mean***	***62′917***	***59′973.7***	**4.91%**	**3′810.66**	**0,24**

## Conclusion

The objective of this study was threefold: first, to examine the behavior of regular donors in Geneva and document the frequency and continuity of their charitable deductions; second, to analyze the relative importance of income and wealth in explaining donor regularity; and third, to apply time-series forecasting to predict future developments in both donation amounts and donor numbers. By addressing these aims with a rare dataset that covers the entire population of Geneva taxpayers between 2001 and 2011, this study provides robust evidence on the dynamics of charitable giving under a tax incentive regime.

The first aim, related to the behavior of regular donors, was particularly motivated by the results shown in Figs. 1 and 2, which reveal a declining pattern in the frequency of deductions both for all donors and specifically for those who donated the legal ceiling of 4. Interestingly, the number of donors who give on a regular basis—between 5 and 11 years during the study period—remains relatively stable. This suggests that the proportion of donors who contribute every other year is almost equivalent to those who donate annually, which makes them particularly relevant to the focus of this study.

Our analysis focused on two subgroups of very regular donors: the *deducters11*, who deduct charitable contributions every year, and the *deducters11-ceiling*, who additionally reach the maximum ceiling of deductions. The *deducters11* group corresponds to 5,948 taxpayers, representing 2.54% of Geneva’s entire taxpayer population in 2001. The central empirical finding is that donor constancy is primarily influenced by wealth rather than income, even though tax incentives are linked to income. In fact, 95% of regular *deducters11* did not reach the ceiling of deductible contributions, indicating that while they use the deduction, their giving is not structured around maximizing the tax incentive. Although there was a temporary peak in deductions following a reform that increased the ceiling, this effect did not persist. We also compared these groups to the subgroup of irregular *deductors* identified in Lideikyte-Huber and Pittavino (2022), who appear to be motivated by tax incentives but do not display regular giving behavior. Additional criteria also emerges regular giving is more common among multi-person households than single taxpayers, and constant givers are, on average, in their mid-50 s to late 60 s, close to retirement age in Switzerland.

Methodologically, we applied three types of regression models. Given the similarity of population characteristics and the limited impact of outliers, standard linear regression using least-squares estimation provided more efficient results than mixed models or robust regression based on M-estimation. This supports the reliability of our regression-based findings.

The results point toward clear policy implications. Since wealth is a critical determinant of consistent giving, a parallel tax incentive linked to wealth—rather than solely income—could strengthen charitable donations. Such a measure, already suggested in legal scholarship (Lideikyte-Huber and Peter 2022), would be in line with the general objectives of Swiss legislation to maximize total giving. Our findings also suggest that household structure and age should be taken into account when designing incentives, given the greater regularity of multi-person households and the concentration of constant donors among taxpayers approaching retirement.

While this article does not formally assess the 2001, 2006, and 2010 tax reforms, their effects on donor behavior have been examined in detail in Lideikyte-Huber and Pittavino (2022). Here, we limit ourselves to descriptive references in order to maintain the paper’s focus on donor regularity, wealth-income interactions, and forecasting perspectives.

In terms of future developments, our forecasts suggest that charitable deductions in Geneva nearly doubled between 2011 and 2021, from CHF 72.7 million to an estimated CHF 137.2 million, compared with CHF 29.1 million in 2001. The number of donors increased almost fourfold over the same period, from 19,335 to about 72,349, very close to the actual figure of 65,574 reported by the Geneva authorities. These results highlight the distinctiveness of our estimates, which focus exclusively on tax-deductible donations. In contrast, broader surveys such as Spendenreport Schweiz (2022) report that 75% of households in French-speaking Switzerland donated in 2021, but our data indicate that only 19% of Geneva taxpayers claimed deductible donations in 2011, underlining the importance of distinguishing between general and tax-deductible giving.

Taken together, these results provide strong empirical evidence that wealth plays a central role in sustained charitable behavior, a factor often neglected in the literature (Wayo and Tinsley 2009). They also demonstrate that constant donors respond only weakly to changes in income-based incentives, underscoring the need to reconsider how tax policy can effectively mobilize private resources for the public good. While this paper remains deliberately empirical and descriptive, it provides a solid evidence base that can inform future modeling efforts. In particular, the parameters on donor regularity and wealth responsiveness established here could serve as the foundation for policy-oriented simulations of alternative tax regimes, including adjustments to deduction ceilings or the design of wealth-linked incentives (Ring and Thoreson 2021, 2022, 2025).

The two components of the study serve distinct but complementary aims: the regression analysis identifies the behavioral drivers of charitable giving, particularly the interaction of income and wealth, while the forecasting analysis offers a forward-looking view of aggregate deduction trends. The link between regression and forecasting is therefore not methodological but conceptual: the regressions highlight the structural role of wealth, while the forecasts trace the likely trajectory of total donations in a reformed tax environment.

By combining rare administrative data, rigorous applied regression-based econometric analysis, and validated forecasts, this study contributes to both the empirical and methodological literature on charitable giving. It offers insights of direct relevance for policymakers, while also establishing a basis for future research that integrates empirical findings into structured policy models aimed at evaluating and optimizing tax incentives for philanthropy.

## Supplementary Information

Below is the link to the electronic supplementary material.

Supplementary file1 (PDF 335 KB)

## Data Availability

The data that support the findings of this study are available from Tax Administration of the Canton of Geneva (TACG). Restrictions apply to the availability of these data, which were used under license for this study. Data are available from the author(s) with the permission of Tax Administration of the Canton of Geneva (TACG).
