# Supplementary material for: Modeling Geneva charitable deductions: regular giving and future trends
Source: Stat Methods Appt. 2026 Jan 19;35(1):129–60. doi: 10.1007/s10260-025-00828-7 (PMC13171932; doi:10.1007/s10260-025-00828-7)
Supplement: Supplementary file 1 — Supplementary file1 (PDF 335 KB) [file 10260_2025_828_MOESM1_ESM.pdf]

Supplementary Material for "Modeling Geneva Charitable Deductions: Regular Giving and Future Trends"

1. Robustness tests for the non-linear and moderating effects of income

Robustness checks were conducted to test the inclusion of the interaction effect, for potential non-linear effects, model misspecification and model selection.

(a) *Interaction effects.*
The nested-model tests, reported in Table SM1 and summarized in Table 6 of the main text, indicate that the income × wealth interaction significantly improves model fit in both OLS and random-intercept mixed-effects models ($F = 1352$, $p < 0.001$; $\chi^2 = 517$, $p < 0.001$). This interaction captures a clear moderating role of wealth: conditional on wealth, the negative marginal coefficient of income is attenuated, suggesting that higher wealth offsets the negative association between income and deductible donations.

Table SM1. Table showing the results from the test for interaction of the nested models: standard linear (OLS) and linear mixed models (LME).

Models	Random Structure	Interaction	Test	p-value	Result
**OLS**	**–**	**Yes**	**F = 1352**	**< 0.001**	**Better fit**
LME[1]	Random intercept: (1 | ID)	Yes	$\chi^2 = 517$	< 0.001	Better fit
LME[2]	Random slope: (Year |ID)	Yes	$\chi^2 = 0$	=1	

[1]LME: Linear Mixed-Effects Model with a random intercept.
[2]LME: Linear Mixed-Effects Model with a random slope for years.

(b) *Non-linear effects.*
To assess whether the negative income coefficient could arise from model misspecification, we estimated additional models including quadratic and log-log transformations of income, whose results are reported in Table SM2. In the linear and mixed-effects specifications, the squared term of income ($\beta^2_{Inc} < 0$, $p < 0.05$) is negative and significant, indicating diminishing marginal effects of income on giving - i.e., as income rises, its incremental contribution to charitable deductions declines. Log-log models yielded consistent but weaker patterns, confirming that the effect of income becomes progressively flatter or slightly negative at higher income levels. Results confirm that the squared income term (β^2Inc) remains negative and significant across specifications, indicating diminishing marginal effects.

Table SM2. Table showing the non-linear effects of income β^2Inc on the classic linear model (OLS), on the linear mixed-effect (LME) models and on the log-log linear model (log-log) with interaction with wealth.

Models	βInc	pInc	β²Inc	pInc	βWth	pWth	βInt	pInt	R2Adj
**OLS**	8.9x10-3	<0.05	**-3.8x10-10**	<0.05	2.8x10-4	<0.05	4.8x10-11	<0.05	0.34
**LME[1]**	*4.1e-03*	<0.05	**-3.2x10-10**	<0.05	6.5x10-4	<0.05	4.1x10-11	<0.05	0.50
**LME[2]**	*4.2x10-3*	<0.05	**-3.2x10-10**	<0.05	6.4x10-4	<0.05	4.1x10-11	<0.05	0.62
**Log-log[3]**	*-0.25*	*<0.05*	-	-	*-0.51*	*<0.05*	0.05	*<0.05*	0.20
**Log-log[4]**	***-0.11***	***0.11***	***-0.01***	***0.03***	*-0.56*	*<0.05*	0.06	*<0.05*	0.20

Notes: All coefficients significant at $p < 0.05$ unless otherwise indicated.
R^2Adj refers to the adjusted R^2 for OLS and the conditional R^2 for LME models.

[1]LME: Linear Mixed-Effects Model with a random intercept.
[2]LME: Linear Mixed-Effects Model with a random slope for years.
[3]Log-log linear model without interaction.
[4]Log-log linear model with interaction.

(c) Model selection.
Although mixed-effects and robust regressions reduced standard errors, they did not increase explained variance, with adjusted R^2 values ranging between 0.30 and 0.34. Given the homogeneity of the *deducters11* population across years, as reported in Tables 4 and 5 of the main text, random or robust structures provide little added value. Consequently, *the OLS model with the income × wealth interaction was retained as the most parsimonious and interpretable specification*.
Taken together, *these results confirm that wealth is the main structural driver of charitable giving*, while income exhibits a diminishing and partially moderated effect once wealth and tax-ceiling constraints are considered, reinforcing the behavioral interpretation that income effects are bounded by structural fiscal limits.

2. Diagnostic tests in regression models

Specific diagnostic tests for heteroskedasticity (e.g., Breusch–Pagan), checks for model misspecification (e.g., RESET), and plots of the residuals have been conducted to test for the key diagnostics error structure. Table SM3 reports the results of the diagnostic tests for the OLS linear regression models with and without interaction reported in the first row of Table 6 and for all the years under study of the main manuscript. As expected for large administrative data, diagnostic tests indicated eteroskedasticity and departures from normality. These tests justify the use of heteroskedasticity-robust standard errors (HC1) reported in Table SM4.
While for the subset *deducters11-ceiling*, further investigation has not been performed, both due to the perfect fit resulting from the specific tax reform constraint applied, and because it falls outside the primary scope of this study.

Table SM3. Diagnostic tests are reported for the OLS linear models with and without interaction.

**Models**	**Breusch-Pagan**	**df**	**p-value**	**NCV Test χ^2**	**p-value**	**RESET F**	**df1, df2**	**p-value**	**Decision**
**All years[1]**	6122.2	3	< 2.2e-16	35,281,314	< 2.2e-16	1835.50	2, 65422	< 2.2e-16	Robust SE
**All years[2]**	3679.3	2	< 2.2e-16	26,875,551	< 2.2e-16	1304.20	2, 65423	< 2.2e-16	Robust SE
**2001[1]**	2401.2	3	< 2.2e-16	2,353,709	< 2.2e-16	52.00	2, 5942	< 2.2e-16	Robust SE
**2001[2]**	1587.0	2	< 2.2e-16	1,933,987	< 2.2e-16	272.32	2, 5943	< 2.2e-16	Robust SE
**2002[1]**	2066.8	3	< 2.2e-16	1,277,914	< 2.2e-16	43.67	2, 5942	< 2.2e-16	Robust SE
**2002[2]**	1891.3	2	< 2.2e-16	1,251,526	< 2.2e-16	142.4	2, 5943	< 2.2e-16	Robust SE
**2003[1]**	1923.5	3	< 2.2e-16	279,744	< 2.2e-16	271.39	2, 5942	< 2.2e-16	Robust SE
**2003[2]**	1141.2	2	< 2.2e-16	270,097	< 2.2e-16	13.48	2, 5943	1.44e-06	Robust SE
**2004[1]**	2306.6	3	< 2.2e-16	1,085,854	< 2.2e-16	58.899	2, 5942	< 2.2e-16	Robust SE
**2004[2]**	1996.7	2	< 2.2e-16	1,067,712	< 2.2e-16	134.48	2, 5943	< 2.2e-16	Robust SE
**2005[1]**	2955.8	3	< 2.2e-16	1,935,728	< 2.2e-16	344.91	2, 5942	< 2.2e-16	Robust SE
**2005[2]**	1981.8	2	< 2.2e-16	1,698,630	< 2.2e-16	1035.0	2, 5943	< 2.2e-16	Robust SE
**2006[1]**	1824.9	3	< 2.2e-16	428,663	< 2.2e-16	28.684	2, 5942	4.00e-13	Robust SE
**2006[2]**	988.8	2	< 2.2e-16	363,520	< 2.2e-16	251.97	2, 5943	< 2.2e-16	Robust SE
**2007[1]**	3418.8	3	< 2.2e-16	1,268,221	< 2.2e-16	161.69	2, 5942	< 2.2e-16	Robust SE
**2007[2]**	2901.5	2	< 2.2e-16	1,246,098	< 2.2e-16	82.91	2, 5943	< 2.2e-16	Robust SE
**2008[1]**	4478.0	3	< 2.2e-16	3,090,818	< 2.2e-16	1511.7	2, 5942	< 2.2e-16	Robust SE
**2008[2]**	2557.7	2	< 2.2e-16	2,091,930	< 2.2e-16	444.55	2, 5943	< 2.2e-16	Robust SE
**2009[1]**	4687.8	3	< 2.2e-16	4,349,422	< 2.2e-16	841.83	2, 5942	< 2.2e-16	Robust SE
**2009[2]**	2133.5	2	< 2.2e-16	2,717,284	< 2.2e-16	346.61	2, 5943	< 2.2e-16	Robust SE
**2010[1]**	1969.6	3	< 2.2e-16	2,242,309	< 2.2e-16	659.20	2, 5942	< 2.2e-16	Robust SE
**2010[2]**	1277.2	2	< 2.2e-16	1,763,319	< 2.2e-16	258.51	2, 5943	< 2.2e-16	Robust SE
**2011[1]**	381.41	3	<2.2e-16	694,041.9	<2.22e-16	151.74	2, 5942	<2.2e-16	Robust SE
**2011[2]**	279.07	2	<2.2e-16	554,604.5	<2.22e-16	166.32	2, 5943	<2.2e-16	Robust SE

[1] OLS models with interaction with classic standard error.
[2] OLS models without interaction with classic standard error.

Table SM4. Coefficient estimates with related p-values and standard errors for the OLS linear models estimated both with the classic and robust standard errors for the entire 11 years under study and for each year (2001-2011) separately. Robust standard errors (HC1) account for heteroskedasticity detected in diagnostic tests. Interaction terms are retained for theoretical consistency, despite limited statistical significance.

	**OLS linear models with classic and robust standard error**								
**Deducters11**	**βInc**	**pInc**	**SE**	**βWth**	**pWth**	**SE**	**βInt**	**pInt**	**SE**
**All years[1]**	-4.3×10^{-3}	<0.05	2.9×10^{-4}	**$9.2x10^{-4}$**	**<0.05**	1.2×10^{-5}	$1.9x10^{-11}$	<0.05	5.1×10^{-13}
**All years[2]**	4.4×10^{-3}	<0.05	1.7×10^{-4}	**$9.7x10^{-4}$**	**<0.05**	1.3×10^{-5}	-	-	-
**All years[3]**	-4.3×10^{-3}	**0.50**	*6.4×10^{-3}*	**$9.2x10^{-4}$**	**<0.05**	*2.5×10^{-4}*	$1.9x10^{-11}$	**0.19**	1.4×10^{-11}
**All years[4]**	*4.4×10^{-3}*	*0.09*	*2.7×10^{-3}*	**$9.7x10^{-4}$**	**<0.05**	*2.8×10^{-4}*	-	-	-
**2001[1]**	-2.2×10^{-3}	<0.05	4.2×10^{-4}	4.7×10^{-4}	<0.05	2.8×10^{-5}	2.5×10^{-11}	<0.05	9.9×10^{-13}
**2001[2]**	*-2.2×10^{-3}*	*0.57*	3.8×10^{-3}	4.7×10^{-4}	0.06	2.5×10^{-4}	*2.5×10^{-11}*	*0.17*	*1.8×10^{-11}*
**2002[1]**	2.8×10^{-3}	<0.05	4.9×10^{-4}	4.1×10^{-4}	<0.05	2.7×10^{-5}	2.2×10^{-11}	<0.05	1.1×10^{-12}
**2002[2]**	*2.8×10^{-3}*	*0.62*	5.5×10^{-3}	4.1×10^{-4}	0.12	2.6×10^{-4}	*2.2×10^{-11}*	*0.23*	*1.8×10^{-11}*
**2003[1]**	5.3×10^{-3}	<0.05	2.8×10^{-4}	3.2×10^{-4}	<0.05	1.2×10^{-5}	-1.5×10^{-12}	<0.05	5.8×10^{-13}
**2003[2]**	*5.3×10^{-3}*	*0.08*	3.1×10^{-3}	3.2×10^{-4}	<0.05	1.6×10^{-4}	*-1.5×10^{-12}*	*0.83*	*7.0×10^{-12}*
**2004[1]**	4.4×10^{-3}	<0.05	3.7×10^{-4}	2.9×10^{-4}	<0.05	1.6×10^{-5}	1.2×10^{-11}	<0.05	7.6×10^{-13}
**2004[2]**	*4.4×10^{-3}*	*0.28*	4.0×10^{-3}	2.9×10^{-4}	<0.05	1.5×10^{-4}	*1.2×10^{-11}*	*0.35*	*1.3×10^{-11}*
**2005[1]**	*-2.8×10^{-3}*	<0.05	4.1×10^{-4}	2.7×10^{-4}	<0.05	1.7×10^{-5}	3.7×10^{-11}	<0.05	8.3×10^{-13}
**2005[2]**	*-2.8×10^{-3}*	0.53	4.6×10^{-3}	2.7×10^{-4}	0.09	1.6×10^{-4}	3.7×10^{-11}	0.03	1.7×10^{-11}
**2006[1]**	6.8×10^{-3}	<0.05	2.6×10^{-4}	4.5×10^{-4}	<0.05	1.1×10^{-5}	*$-9.7x10^{-12}$*	<0.05	4.4×10^{-13}
**2006[2]**	6.8×10^{-3}	<0.05	3.3×10^{-3}	4.5×10^{-4}	<0.05	1.5×10^{-4}	*$-9.7x10^{-12}$*	*0.07*	*5.4×10^{-12}*
**2007[1]**	2.9×10^{-3}	<0.05	2.4×10^{-4}	3.5×10^{-4}	<0.05	1.2×10^{-5}	3.0×10^{-12}	<0.05	7.3×10^{-12}
**2007[2]**	*2.9×10^{-3}*	*0.34*	*3.0×10^{-3}*	3.5×10^{-4}	<0.05	1.6×10^{-4}	*3.0×10^{-12}*	*0.68*	*3.8×10^{-13}*
**2008[1]**	-6.9×10^{-3}	<0.05	5.2×10^{-4}	7.7×10^{-4}	<0.05	2.7×10^{-5}	2.3×10^{-11}	<0.05	8.4×10^{-13}
**2008[2]**	*-6.9×10^{-3}*	*0.34*	*7.1×10^{-3}*	7.7×10^{-4}	<0.05	3.2×10^{-4}	2.3×10^{-11}	*0.29*	2.1×10^{-11}
**2009[1]**	-5.4×10^{-3}	<0.05	2.3×10^{-3}	7.5×10^{-4}	<0.05	7.5×10^{-5}	8.3×10^{-11}	<0.05	3.7×10^{-12}
**2009[2]**	*-5.4×10^{-3}*	*0.89*	*4.0×10^{-2}*	*7.5×10^{-4}*	*0.37*	*8.3×10^{-4}*	*8.3×10^{-11}*	*0.46*	*1.1×10^{-10}*
**2010[1]**	*$-1.9x10^{-2}$*	<0.05	1.8×10^{-3}	1.9×10^{-3}	<0.05	5.1×10^{-5}	3.7×10^{-11}	<0.05	2.9×10^{-12}
**2010[2]**	*$-1.9x10^{-2}$*	*0.45*	*2.5×10^{-2}*	1.9×10^{-3}	<0.05	8.5×10^{-4}	*3.7×10^{-11}*	*0.54*	*6.0×10^{-11}*
**2011[1]**	1.3×10^{-2}	<0.05	9.0×10^{-4}	5.3×10^{-4}	<0.05	2.6×10^{-5}	*-1.9×10^{-11}*	<0.05	1.6×10^{-12}
**2011[2]**	1.3×10^{-2}	0.06	6.9×10^{-3}	5.3×10^{-4}	<0.05	3.5×10^{-4}	*-1.9×10^{-11}*	*0.280*	*1.5×10^{-11}*

[1] OLS models with interaction with classic standard error.
[2] OLS models without interaction with classic standard error.
[3] OLS models with interaction with robust standard error.
[4] OLS models without interaction with robust standard error.

3. Stationarity tests for ARIMA model

Stationarity for the donors time series was assessed using Augmented Dickey–Fuller (ADF) and KPSS tests. The ADF failed to reject the unit-root null hypothesis at the level, first, and second differences, with p-values of 0.19, 0.91, 0.61, respectively (Table SM5). Conversely, the KPSS test rejected stationarity at the level ($p = 0.046$) but not at the first or second differences ($p = 0.09$ and ≥ 0.10). Consistent with the *ndiffs()* heuristic from the R package *forecast*, these results support a non-seasonal differencing order of $d = 2$.
The ARIMA(0,2,0) model was then estimated, and Ljung–Box tests confirmed that the residuals behave as white noise ($X^2 = 3.61$, df = 8, $p = 0.89$; Table SM6), validating the adequacy of the second-order differencing specification.

Table SM5. Stationarity for the donors time-series.

Series	ADF p-value	KPSS p-value	Stationarity conclusion
Level	0.19	0.046	Non-stationary
First differentiation	0.91	0.093	Borderline / not clear
Second differentiation	0.61	≥ 0.10	Stationary

Table SM6. Residual Diagnostics for the ARIMA(0,2,0) for the donors time-series.

X^2	df	p-value	Diagnostics conclusion
3.61	8	0.89	Residuals are white noise
